# Cytocompatible Hydrogels with Tunable Mechanical Strength and Adjustable Swelling Properties through Photo-Cross-Linking of Poly(vinylphosphonates)

**DOI:** 10.1021/acsami.4c07860

**Published:** 2024-10-15

**Authors:** Anton S. Maier, Salma Mansi, Kerstin Halama, Philipp Weingarten, Petra Mela, Bernhard Rieger

**Affiliations:** † Technical University of Munich, Germany, TUM School of Natural Sciences, Department of Chemistry, WACKER-Chair of Macromolecular Chemistry, Lichtenbergstraße 4, 85748 Garching, Germany; ‡ Technical University of Munich, Germany, TUM School of Engineering and Design, Department of Mechanical Engineering, Chair of Medical Materials and Implants, Munich Institute of Biomedical Engineering, Munich Institute of Integrated Materials, Energy and Process Engineering, Boltzmannstraße 15, 85748 Garching, Germany

**Keywords:** catalytic polymerization, thiol−ene click reaction, hydrogel, oscillatory rheology, cytocompatibility

## Abstract

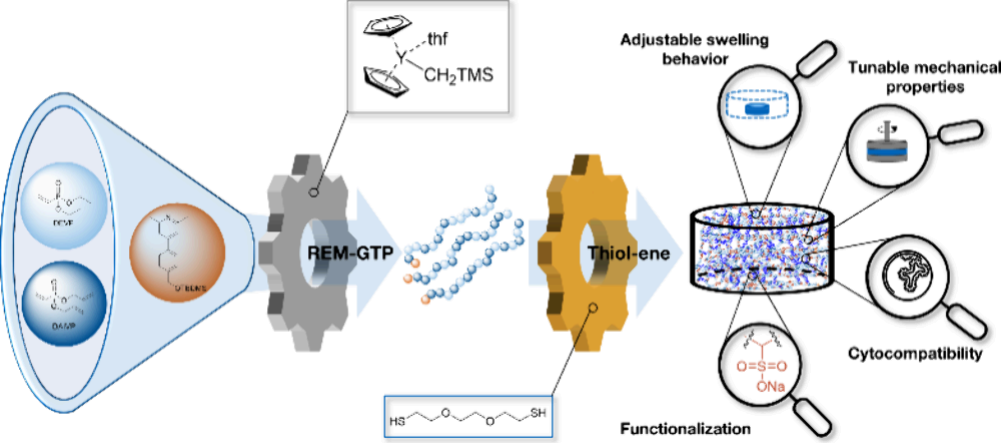

Herein, the synthesis, characterization, and application of a novel synthetic hydrogel based on the photoinitiated cross-linking of poly(vinylphosphonates) is presented. First, statistical copolymers with adjustable ratios of the monomers diallyl vinylphosphonate (DAlVP) and diethyl vinylphosphonate (DEVP), as well as different molecular weights, were obtained via rare earth metal-mediated group-transfer polymerization (REM-GTP) while maintaining narrow polydispersities. The copolymers were cross-linked by applying photoinitiated thiol–ene click chemistry (λ = 365 nm). The network formation was monitored via oscillatory rheology coupled with UV-irradiation, revealing the high spatiotemporal control of the reaction. Moreover, the equilibrium storage moduli of poly(vinylphosphonate)-based hydrogels increased with a growing number of DAlVP units and upon application of a different cross-linker, which was additionally confirmed by nanoindentation experiments. In contrast, the water uptake of hydrogels decreased with higher DAlVP amounts in the corresponding hydrogels due to lower chain mobility and an overall increase in the hydrophobicity of the samples. Upon successful functionalization of P(DEVP-*stat*-DAlVP) copolymers with sodium 3-mercaptopropane-1-sulfonate, as indicated via ^1^H DOSY NMR, the respective cross-linked materials displayed a remarkable increase in the water uptake; thus, presenting highly hydrophilic gels with an apparent interplay between water uptake, cross-linking density, and functionalization degree. Finally, the purified hydrogels showed cytocompatibility and enabled cell adhesion of human umbilical artery smooth muscle cells (HUASMCs) after direct seeding. The materials further allowed the adhesion and growth of an endothelial layer, triggered no pro-inflammatory response as evidenced by cytokine release of M0 macrophages, and exhibited antibacterial properties toward *S. aureus* and *E. coli*.

## Introduction

Hydrogels are a class of materials based on natural or synthetic polymers with the ability to retain significant amounts of water within their cross-linked, three-dimensional network without dissolution.^1^ Depending on the mechanism of network formation, these materials are typically classified as supramolecular hydrogels or chemically cross-linked hydrogels. In supramolecular materials, cross-linking occurs through noncovalent interactions, e.g., ionic or van der Waals interactions, host–guest chemistry, or hydrogen bonding. In contrast, hydrogels are categorized as chemically cross-linked if covalent bonds connect the polymer chains.^2−5^ The outstanding physicochemical properties of hydrogels, such as high water absorption, mechanical stability, biocompatibility, and biodegradability, render them appealing candidates for various biomedical applications.^6−10^ In this context, cross-linked polymer materials are highly relevant in drug delivery,^11−13^ tissue engineering,^14,15^ wound healing,^16,17^ and as antimicrobial coatings.^8,18,19^ Beyond biomedicine, hydrogels have gained significant importance in various other fields, inter alia, in wastewater purification^20,21^ or smart devices like actuators and sensors employing the stimuli-responsiveness (pH, temperature, light) of certain materials.^22−28^ The tunability of the stimuli-responsive properties of hydrogels is one reason why synthetic polymers have emerged as promising candidates for tailored hydrogel applications. Additionally, synthetic polymers allow a facile adjustment of mechanical properties, and swelling behavior and guarantee the reproducibility of hydrogel synthesis. Commonly applied polymers for non-natural hydrogels comprise water-soluble and biocompatible macromolecular compounds, such as poly(ethylene glycol),^29,30^ poly(vinyl alcohol),^31,32^ and poly(acrylate)/poly(acrylamide) derivatives.^33,34^ Another class of synthetic polymers exhibiting good water solubility, high biocompatibility, as well as a lower critical solution temperature (LCST) are poly(vinylphosphonates) obtained via rare earth metal-mediated group-transfer polymerization (REM-GTP).^35−37^ Polymer synthesis through REM-GTP, enabling the polymerization of Michael-type monomers through repeated 1,4-conjugate addition, is particularly interesting for biomedical applications. The reason for this is the precise control over the polymer microstructure while maintaining narrow polydispersities, resulting in highly defined polymeric structures.^38,39^ This is due to the continuous coordination of the growing polymer chain to the catalyst center, leading to the simultaneous growth of polymer chains at the active catalyst molecules. Furthermore, this propagation mechanism enables the synthesis of block copolymers from various types of α,β-unsaturated monomers in accordance with their coordination strength to the catalyst.^40,41^ In contrast, the synthesis of statistical copolymers from various structurally related dialkyl vinylphosphonates is mainly dependent on the steric demand of the growing chain rather than the coordination strength to the catalyst center.^37^ In this respect, the copolymerization of a mixture of diallyl vinylphosphonate (DAlVP) and diethyl vinylphosphonate (DEVP) to statistical copolymers is possible and yields water-soluble polymers susceptible to postpolymerization functionalization of the allyl side groups with a variety of synthetic methods.^42,43^ One common approach for functionalizing allylic and vinylic motifs in polymers with, e.g., biologically active substrates involves the application of thiol–ene click chemistry.^44−46^ The thiol–ene reaction is known for its high reaction rates under mild reaction conditions, high yields, and low to no side products. Moreover, thiol–ene click reactions are very robust, tolerating moisture and oxygen, and are often photoinitiated, allowing high temporal and spatial control over the photochemical initiation.^47^ Finally, thiol–ene click chemistry exhibits high compatibility with a variety of functional groups as well as orthogonality with other common organic reaction types, making it a very versatile tool for introducing functional motifs into vinyl group-containing polymers.^47−50^ Since the beginning of the century, thiol–ene click chemistry has been widely explored in hydrogel synthesis, as it allows the in situ and in vivo formation of soft, tissue-like materials under physiological conditions upon UV-light irradiation. In this context, often biocompatible, PEG- or poly(acrylate)-based polymers with pending vinyl groups are employed in combination with di- or multifunctional thiols for the formation of biodegradable, cross-linked networks with a considerable potential for applications in tissue engineering and drug delivery with specific requirements to the release profile.^19,30,51−55^ In the present study, this concept is transferred to statistical copolymers obtained from DEVP and DAlVP obtained via rare earth metal-mediated group-transfer polymerization (REM-GTP), enabling the synthesis of noncytotoxic hydrogels with tunable mechanical strength. Further, we demonstrate an adjustment of the swelling ratio by control of the cross-linking density and through functionalization of P(DEVP-*stat*-DAlVP) copolymers with sodium 3-mercaptopropane-1-sulfonate via click chemistry prior to cross-linking.

## Results and Discussion

### Copolymerization of Diethyl Vinylphosphonate (DEVP) and Diallyl Vinylphosphonate (DAlVP)

**Scheme 1:**
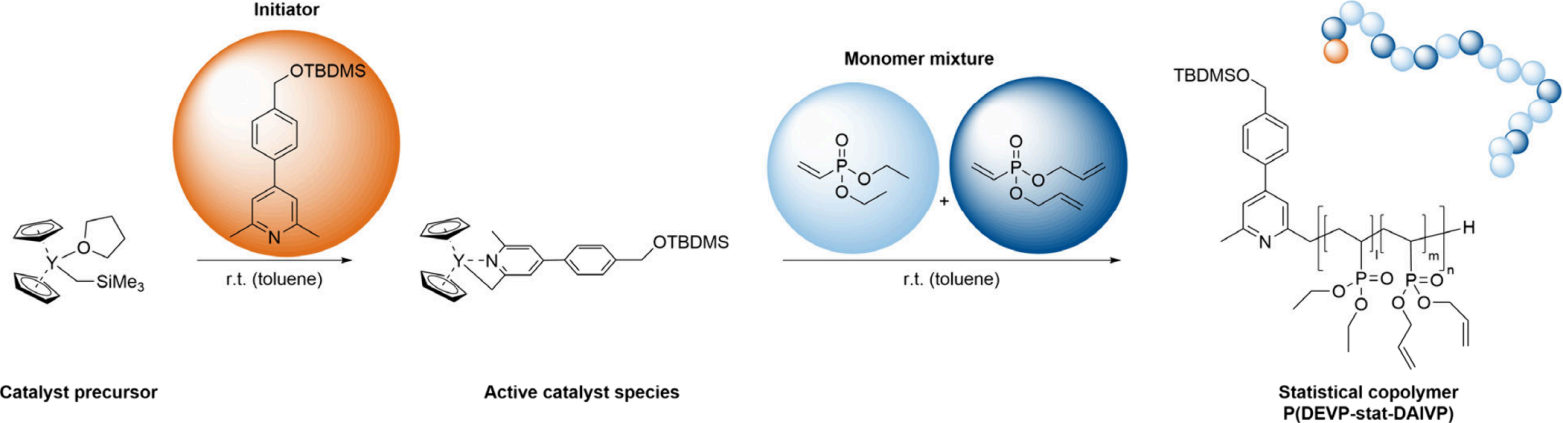
Statistical Copolymerization of DEVP and DAlVP with the CH-Bond Activated Species of Cp_2_YCH_2_TMS(thf)

Prior to the copolymerization of DEVP and DAlVP, in situ CH-bond activation via σ-bond metathesis with equimolar amounts of 4-(4-(((*tert*-butyldimethylsilyl)oxy)methyl)phenyl)-2,6-dimethylpyridine and the catalyst precursor Cp_2_YCH_2_TMS(thf) is conducted to form the initiating complex for subsequent polymerization (Scheme 1).^56−58^ The introduction of the 2,4,6-trimethylpyridine-derivative yields an initiating ligand with lower basicity, thus suppressing undesired deprotonation reactions of the monomers, and further incorporates an end-group into the copolymers, exhibiting distinct OTBDMS-signals in the ^1^H NMR spectrum. This enables the facile estimation of the molecular weight of the respective copolymers via ^1^H NMR spectroscopy, since the determination of the absolute molecular weights of the copolymers using size-exclusion chromatography multiangle light scattering (SEC-MALS) requires the determination of the refractive index increment for each copolymer composition. After quantitative CH-bond activation via ^1^H NMR spectroscopy in C_6_D_6_ is confirmed by the absence of the CH_2_-group signal (doublet, δ = −0.66 ppm) of Cp_2_YCH_2_TMS(thf) (Figure S1), a mixture of the monomers DEVP and DAlVP is added quickly to ensure simultaneous initiation of the copolymerization at all catalyst centers. Conversion of the monomers is monitored via ^31^P NMR spectroscopy in MeOD. Upon quantitative conversion, the polymerization is stopped by the addition of 0.5 mL of undried methanol, and the copolymers are purified (for experimental details, see the Supporting Information).

Polymerization experiments yielded copolymers with a tunable composition by adjusting the monomer feed ratio while maintaining narrow polydispersities (Table 1 and Figures S5–S12). The results in Table 1 demonstrate excellent control over polymer microstructure and chain length.

**Table 1:** Results of the Copolymerization of DEVP and DAlVP with the CH-Bond Activated Species of Cp_2_YCH_2_TMS(thf)

entry	DEVP/DAlVP/Cat.^a^	targeted DAlVP content [%]	*X* [%]^b^	DAlVP content [%]^c^	*M*_n,NMR_^d^	IE^e^	*Đ*^f^
1	180/20/1	10	>99	9.0	39	86	1.08
2	270/30/1	10	>99	9.0	82	61	1.09
3	360/40/1	10	>99	8.8	107	56	1.09
4	450/50/1	10	>99	12.3	173	45	1.24
5	300/100/1	25	>99	23.9	162	37	1.34
6	320/80/1	20	>99	19.7	148	41	1.06
7	340/60/1	15	>99	13.9	200	29	1.06
8	380/20/1	5	>99	5.8	213	29	1.06

a Reactant ratio desired.

b Conversion determined via ^31^P NMR spectroscopy in MeOD.

c Determined via ^1^H NMR spectroscopy by comparison of the CH_2_ signals of DEVP (4.18 ppm, *m* = I/4) and DAlVP (4.63 ppm, *n* = I/4).

d Calculated via ^1^H NMR spectroscopy by comparison of the – OTBDMS signals of the initiator at 0.14 ppm (I = 6H) and the CH_2_ signals of DEVP (4.18 ppm, *m* = I/4) and DAlVP (4.63 ppm, *n* = I/4).

e IE = *M*_n,calc_/*M*_n,NMR_, with *M*_n,NMR_ = 327.54 g/mol + *m* × 164.14 g/mol + *n* × 188.16 g/mol.

f Polydispersity determined via size-exclusion chromatography multiangle light scattering (SEC-MALS) in THF:H_2_O (1:1) with 340 mg/L 2,6-di-*tert*-butyl-4-methylphenol (BHT) and 9 g/L tetra-*n*-butylammonium bromide (TBAB).

Entries 1–4 (Table 1) display a variation of the polymer chain length while targeting the same proportion of cross-linkable DAlVP units in the copolymers. In all cases, the targeted percentage of DAlVP in the copolymers is met with relatively high precision compared with the values derived from the ^1^H NMR spectra. Further, switching toward higher monomer/catalyst ratios, an increase in the molecular weight of the polymers as determined via ^1^H NMR spectroscopy is observed, with only a slight decrease in the initiator efficiency and a minor increase in the polydispersity. However, note that the initiator efficiencies given in Table 1 do not rely on absolute molecular weight determinations. Therefore, the significance of those values is rather low, as molecular weight determination via ^1^H NMR spectroscopy (Figure S2) is prone to errors. Overall, these results demonstrate a straightforward adjustment of the molecular weight of the P(DEVP-*stat*-DAlVP) copolymers by a variation of the monomer/catalyst ratio. Comparing entry 3 to entries 5–8, the results in Table 1 illustrate a tunable copolymer composition by alteration of the monomer feed ratio. This gives access to different polymer microstructures with varying amounts of cross-linking sites. In all cases, successful copolymer formation was confirmed via ^31^P NMR and ^1^H DOSY NMR spectroscopy (Figures S3 and S4). To reduce catalyst depletion and facilitate handling of the polymers (short-chain polymers exhibit higher tack), we decided to proceed with higher molecular weight polymers (Table 1, entries 3 and 5–8) for the hydrogel synthesis. In addition to these results, the thermal properties of these novel copolymers were thoroughly investigated to obtain a more detailed understanding of this type of poly(vinylphosphonates). In accordance with previous reports, the copolymers synthesized in this study exhibit fully reversible lower critical solution temperature (LCST) behavior within the physiological temperature range. In this context, an aqueous solution of polymer 1 undergoes coil–globule transition at 36 °C, which was detected by measuring the transmittance of the solution as shown in Figure S13.^37^ Further, thermogravimetric analysis of entry 3 yielded a thermal decomposition onset of 313 °C (Figure S14), matching the values found in studies on the thermal behavior of poly(vinylphosphonates) very well.^59^ Finally, the differential scanning calorimetry (DSC) results presented in Figure S15 revealed the absence of a melting point in P(DEVP-*stat*-DAlVP), confirming that the statistical copolymerization of DEVP and DAlVP with Cp_2_YCH_2_TMS(thf) proceeds in a stereoirregular fashion, yielding atactic, amorphous polymers.^60^

### Hydrogel Formation and Rheological Characterization

**Scheme 2:**
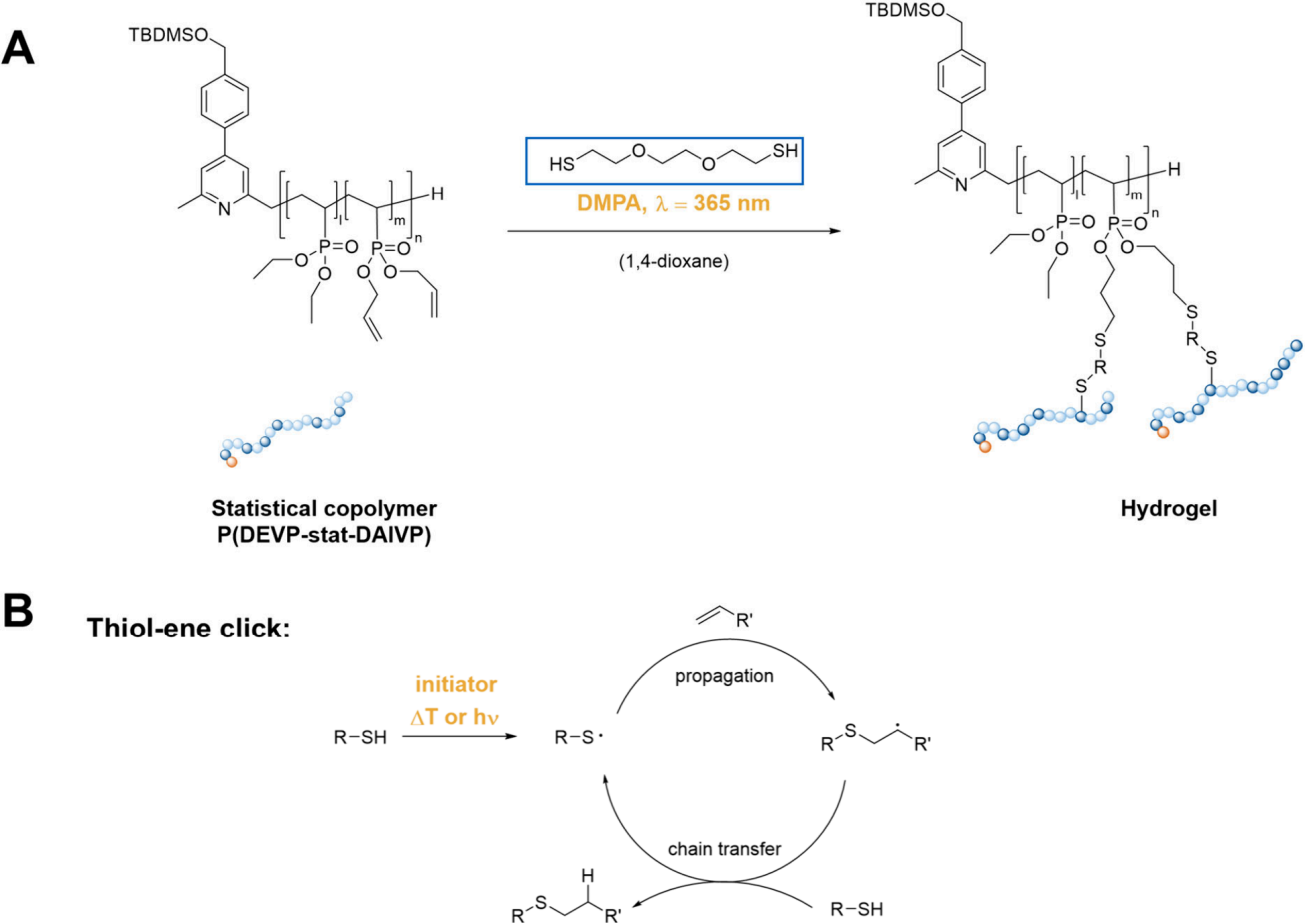
(A) Schematic Representation of the Cross-Linking of P(DEVP-*stat*-DAlVP) Copolymers via Photoinitiated Thiol–ene Click Reaction, Applying 2,2-Dimethoxy-2-phenylacetophenone (DMPA) as the Photoinitiator and 3,6-Dioxa-1,8-octanedithiol (blue) as Cross-Linker; (B) Underlying Reaction Mechanism of the Thiol–ene Click Reaction

**Figure 1:**
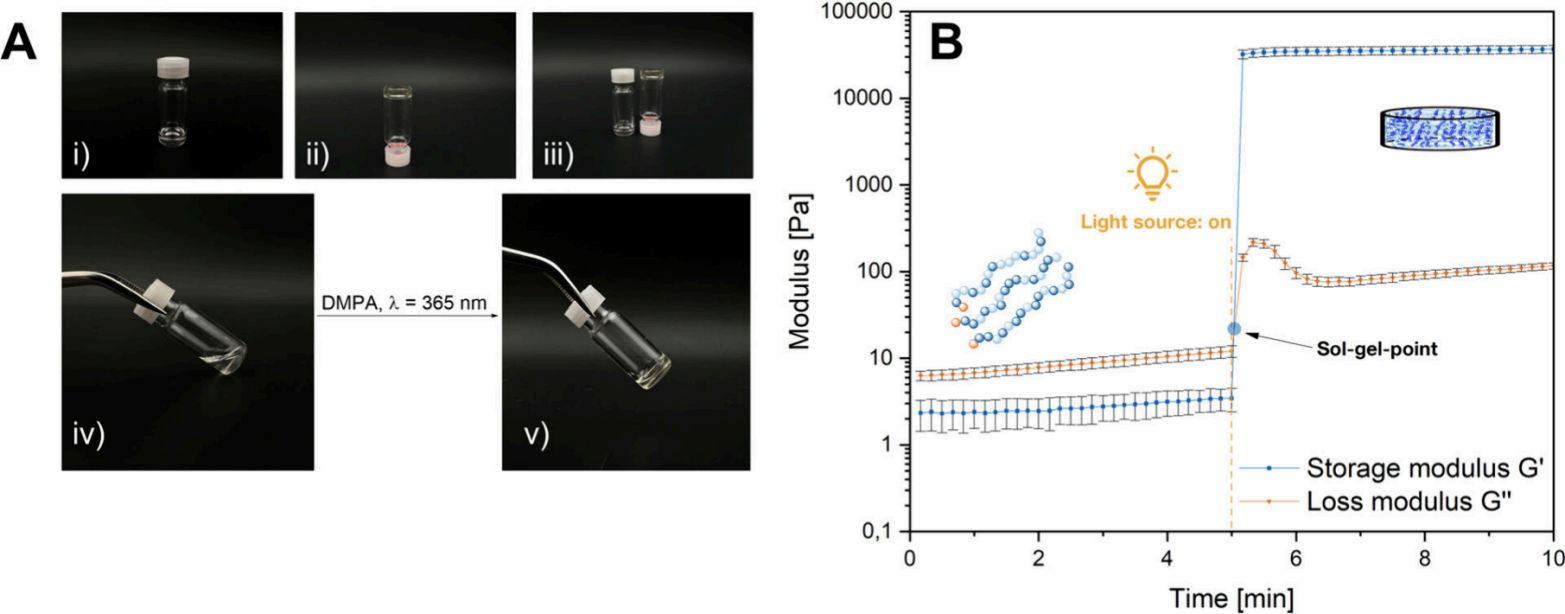
(A) Cross-linking reaction of P(DEVP-*stat*-DAlVP) with 3,6-dioxa-1,8-octanedithiol using UV-light (λ = 365 nm) initiated thiol–ene click chemistry and 2,2-dimethoxy-2-phenylacetophenone (DMPA) as the photoinitiator. (i) and (iv): Samples in liquid state; (ii) and (v): Cross-linked samples; and (iii): Comparison of solution and gel. (B) Rheological investigation of gelation process: time sweep with the experimentally determined values for the deformation and frequency (γ = 1%, *f* = 5 Hz), demonstrating the kinetics of the thiol–ene-mediated cross-linking reaction of P(DEVP-*stat*-DAlVP).

To explore hydrogel formation originating from P(DEVP-*stat*-DAlVP) copolymers upon application of the thiol–ene click reaction, commercially available, PEG-based 3,6-dioxa-1,8-octanedithiol was selected as a model cross-linker for initial experiments. Those experiments involved testing different solvents, reaction conditions, polymer concentrations, and a variation of the curing procedure (Table S1). Concerning the solvent, a mixture of tetrahydrofuran and methanol was selected for the first experiments following reports from Rieger et al.^42^ Additionally, water and 1,4-dioxane were tested in the cross-linking reaction, leading to successful hydrogel formation under photochemical reaction conditions with 2,2-dimethoxy-2-phenylacetophenone (DMPA) as an initiator even in the presence of oxygen. This demonstrates the broad applicability and robustness of the thiol–ene click reaction, as already discussed in the introduction.^47−50^ Considering the choice of solvent for the hydrogel syntheses presented in Table S1, water was not selected due to the surface-active properties of the P(DEVP-*stat*-DAlVP) polymers, causing foaming upon polymer dissolution and leading to inclusions of air bubbles in the cross-linked materials. As this caused poor reproducibility of the synthesis and decreased structural integrity, more focus was placed on the THF/MeOH-mixture and 1,4-dioxane. Both dissolved the polymers equally well, leading to homogeneous solutions and, accordingly, smooth hydrogels. To facilitate experimentation and avoid toxic MeOH, 1,4-dioxane was selected as a solvent instead of the mixture. Further, different polymer concentrations were tested for the cross-linking reaction. Despite successful hydrogel synthesis among all tested concentrations, high polymer concentrations were selected for the standardized synthesis procedure, as this should ideally favor the desired intermolecular cross-linking reaction over intramolecular thiol–ene click reactions of the allyl group-containing poly(vinylphosphonates). Finally, a thermally initiated thiol–ene click reaction with azobis(isobutyronitrile) (AIBN) was compared to the photochemical process applying DMPA. While both reactions resulted in hydrogel formation, the photochemical reaction was preferred because it allows a more detailed study of the sol–gel transition (gelation process) via oscillatory rheology, enabling a high spatial and temporal control over the click reaction by selectively switching on UV-light irradiation (Scheme 2 and Figure 1).^47^

To obtain a profound understanding of the gelation process, the limits of the linear viscoelastic region (LVR), in which nondestructive rheological testing due to proportionality of the shear strain and the shear stress is guaranteed, are determined by a series of rheological experiments. Further, the application of oscillatory rheology within the LVR ensures comparability of the results obtained for different samples by applying the same measurement frequency and amplitude. The preliminary experiments for evaluating the limits of the LVR consist of four steps:^61^ (1) a time sweep with arbitrary frequency and amplitude to determine the rate of gel formation, (2) a deformation sweep on a fully gelled sample with arbitrary frequency to determine the LVR region with regard to the strain, (3) a frequency sweep with the determined amplitude from (2) on a cross-linked sample to evaluate the linear equilibrium modulus plateau of the hydrogel, and (4) a time sweep to obtain the gelation kinetics and the equilibrium moduli with the previously obtained values for amplitude and frequency (Figure 1B). Step (1), however, was not carried out as a rheological experiment, as the initial experiments on hydrogel formation already indicated immediate hydrogel formation upon irradiation. Consequently, the deformation sweep (Figure S18) and subsequent frequency sweep (Figure S19), both performed on fully cross-linked samples, were conducted after irradiation of the polymer solution for 5 min.

For rheological experiments, the hydrogels were formed between the rheometer plates in situ. Therefore, a polymer solution containing photoinitiator and cross-linker was applied onto a glass plate at the rheometer, and the upper plate was lowered, leading to a quantitative filling of the gap. Subsequently, gelation was initiated at the desired time by irradiating the sample through the transparent glass plate at 365 nm from below (for experimental details, see the Supporting Information). The deformation sweep illustrated in Figure S18 indicated linear behavior of the storage modulus *G*′ and the loss modulus *G*′′ for deformations between 0.1% and 1%. Thus, an amplitude of 1% was selected for the subsequent frequency sweep (Figure S19), in which a low-frequency plateau of the values for *G*′ and *G*′′ appeared between 0.1 and 10 Hz. For conducting time-resolved small-amplitude oscillatory shear experiments, the testing frequency must be within this window.^61^ Therefore, a frequency of 5 Hz along with the deformation of 1% was applied in a time-resolved rheological experiment, in which gelation was initiated by irradiation after five min (Figure 1B). As evidenced by the results, the samples exhibited liquid-like behavior before irradiation (*G*′ < *G*′′), whereas immediate cross-linking (*t* < 3 s) was observed (*G*′ > *G*′′) after activating the light source with an intensity of 150 mW cm^–2^ (Figures 1B and S20). This demonstrates the excellent control over the thiol–ene click reaction and its high reaction rates as previously reported.^47−50^ Studying the kinetics of the thiol–ene reaction by monitoring the storage moduli revealed a correlation between the gelation rate and light power intensity (Figure S20). However, we believe the short gelation times obtained in this study are mainly attributed to the high polymer concentrations (333 mg mL^–1^) and the excess cross-linker used. Considering the behavior of the moduli in Figure 1B upon UV-irradiation, *G*′ undergoes a stepwise transition, whereas *G*′′ exhibits a maximum, suggesting a rapid transition of the viscous liquid into a glassy state. This behavior is described as vitrification (transition into a glassy state) and is accompanied by a simultaneous peak in the loss factor tan δ (ratio between *G*′′ and *G*′) illustrated in Figure S21.^62,63^ As the measurement proceeded, *G*′′ and tan δ decreased toward nearly constant plateau values, indicating a decrease in the viscous portion of the complex modulus G*. Regarding the radical reaction mechanism presented in Scheme 2B, this behavior is likely attributed to the ongoing radical reaction causing a postcuring after initiation until a constant, quantitative cross-linking degree is reached, indicated by the stable *G*′′ and tan δ values. Control experiments without irradiation and without the addition of an initiator revealed no gel formation (Figures S23 and S24), whereas the cross-linking process in the absence of a cross-linker led to the slow formation of a weak gel (Figure S25). Therefore, only a minor contribution of the formation of C–C bonds via olefin coupling is proposed, as the kinetic curve presented in Figure 1B in the presence of cross-linker not only exhibits significantly higher reaction rates of the C–S coupling but also results in the formation of a stronger gel as indicated by the values of *G*′. Figure 1B also demonstrates excellent reproducibility of the experiments, as indicated by the small error bars, justifying the previously determined frequency and amplitude. Furthermore, the experiments shown by the rheological results in Figure 1B serve as proof of concept for hydrogel synthesis via cross-linking of P(DEVP-*stat*-DAlVP) with 3,6-dioxa-1,8-octanedithiol. In addition to the findings presented in Figure 1, a comparison of the infrared spectra of the cross-linker, a polymer (Table 1, Entry 1), and the corresponding hydrogel indicated a successful photochemical cross-linking of poly(vinylphosphonates) with dithiols. This was confirmed in the hydrogel spectrum by the absence of characteristic thiol stretching bands (2550 cm^–1^) and the appearance of CH_2_–O–CH_2_ asymmetric stretching bands (1180–1060 cm^–1^) inherent to the PEG-chain of the cross-linker (Figure S16).^64^ Thermogravimetric analysis of water-swollen hydrogels displayed dehydration, as shown in Figure S17, followed by the transitions already observed for the polymers (Figure S14).

### Tuning of Mechanical Strength

**Figure 2:**
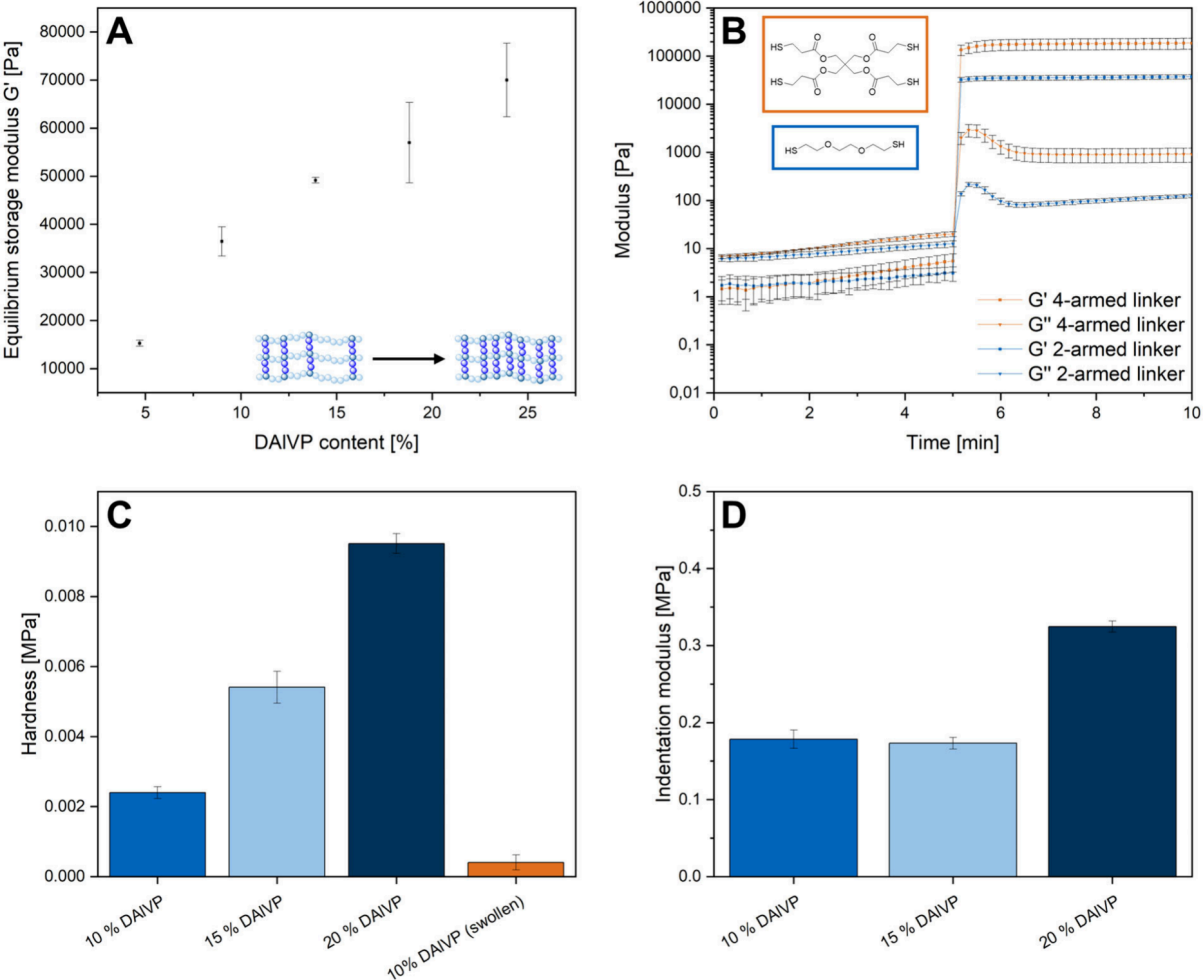
Rheological investigation of the mechanical properties of corresponding hydrogels for different copolymer compositions (Table 1, entries 3 and 5–8) (A) and different cross-linkers (B) at standardized measurement conditions of 5 Hz frequency and 1% deformation. Mechanical properties were obtained through nanoindentation of various samples: Hardness of dried samples compared to a swollen hydrogel sample (C) and indentation moduli of different dry samples (D) with respect to the amount of cross-linkable DAlVP units in the corresponding polymers (Table 1, entries 3, 7, and 6).

After the successful application of oscillatory rheology to characterize the gelation kinetics, it was further selected as a tool for evaluating the mechanical strength of hydrogels. The mechanical properties of hydrogels are mainly affected by the cross-linking density, which in turn can be influenced by various factors, such as type of cross-linker or ion concentrations.^65,66^ We selected two approaches for the variation of viscoelastic properties, both aiming at an alteration of the cross-linking density or the mesh size, respectively. First, the effect of the copolymer composition on the rheological results of the corresponding hydrogels was investigated. In this context, copolymers with similar molecular weights but different amounts of cross-linkable DAlVP units (see Table 1, entries 3 and 5–8) were compared when cross-linked with the model cross-linker 3,6-dioxa-1,8-octanedithiol. Oscillatory rheology was conducted under the previously determined, standardized measurement conditions with a frequency of 5 Hz and a deformation of 1% (Figures 1B, S18, and S19). Further, identical ratios of cross-linker and photoinitiator with respect to the number of cross-linkable units in the copolymers were applied to ensure comparability of the results. Measurements were performed at least in triplicate, and the plateau values of the storage modulus for each kinetic curve were used as a measure of the mechanical strength (for the kinetic curves of each copolymer composition, see the Supporting Information, Figure S22). Figure 2A displays an increase in the mechanical strength of the hydrogels with an increasing number of DAlVP units in the copolymers. This reflects the expected trend, as only DAlVP can undergo a thiol–ene click reaction with the cross-linker. Switching to higher amounts of cross-linkable units (Table 1, entry 5), premature gelation was observed with high reproducibility (Figure S22). This was attributed to the initiation of the cross-linking process by incident light or oxygen diradicals, which is more pronounced if a higher number of cross-linking sites is available and, therefore, is reflected in the increase of moduli during rheological measurements despite light exclusion. However, these results demonstrate tunability of the mechanical properties of the corresponding hydrogels upon adjustment of the monomer feed ratio during polymerization, only limited by premature gelation and decreasing copolymer solubility in polar solvents with increasing DAlVP contents. As a second approach for adjusting the mechanical properties of these novel materials, the cross-linker applied during hydrogel synthesis is easily exchangeable with an impact on the resulting material characteristics. This was demonstrated by performing time-resolved rheological measurements with the same polymer (Table 1, entry 3) but testing different cross-linkers. Figure 2B shows a comparison of the rheological data obtained for the model cross-linker 3,6-dioxa-1,8-octanedithiol (blue curve) and pentaerythritol-tetrakis(3-mercaptopropionate) (orange curve) upon application of equimolar amounts of SH-groups. In other words, only half of the concentration of the 4-armed linker was applied compared to the dithiol to assess the effect of the linker structure exclusively. These measurement results reveal that besides the number of cross-linking sites in the polymer, also the functionality of the linker plays a crucial role with respect to the resulting mechanical properties of the gels. Whereas the model cross-linker is only bifunctional (two thiol groups), the four-armed linker allows for the formation of a more densely cross-linked network, which is reflected in higher values of *G*′ and *G*′′ and corresponds to a higher mechanical strength. Thus, the choice of cross-linker allows for an adjustment of the mechanical properties of materials cross-linked via thiol–ene click reactions. In addition to the rheological characterization of hydrogels, nanoindentation was applied to determine the (surface) mechanical properties of the cross-linked materials. In this context, different dried samples originating from polymers with a rising number of DAlVP units (Table 1, entries 3, 7, and 6) and an increasing cross-linking density were investigated. Regarding the hardness of each sample, higher numbers of DAlVP units in the copolymers resulted in an increased hardness of the cross-linked materials (Figure 2C). Additionally, we observed similar trends regarding the indentation modulus of the samples. Whereas the covalent networks of polymers with 10% and 15% DAlVP units exhibited similar indentation moduli, the sample with the highest cross-linking degree (20% DAlVP) revealed a significantly higher stiffness (Figure 2D). Comparing the water-swollen state of the hydrogels to their dry state, nanoindentation of an exemplary hydrogel revealed a drastically decreased sample hardness after swelling in water. Considering the apparent structure–property relationships between the copolymer structure and the hydrogel properties found for the swelling behavior (discussed in the next section), the mechanical properties determined via rheology (Figure 2A), and the dry sample hardness (Figure 2C), the trend in the mechanical properties is likely to persist in the swollen hydrogels.

### Water Uptake

**Figure 3:**
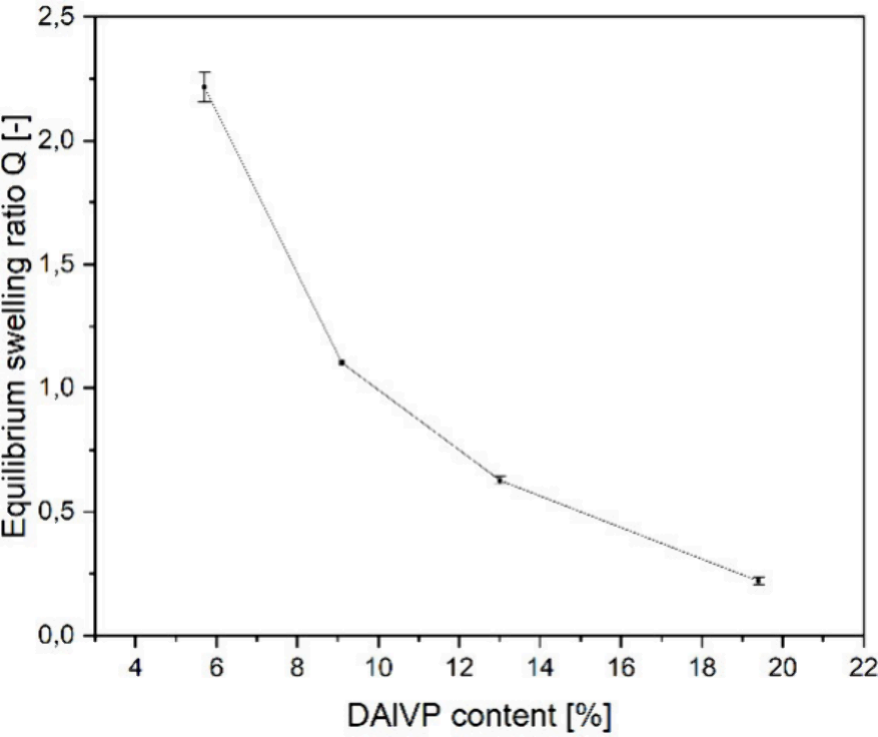
Equilibrium swelling ratio of different hydrogels with respect to the corresponding amounts of DAlVP in the P(DEVP-*stat*-DAlVP) copolymers (Table 1, entries 3 and entries 6–8).

The water uptake of poly(vinylphosphonate)-based hydrogels was investigated by comparing the weight of hydrogels after drying in vacuo overnight with the weight after immersing the samples in water for 6 h, ensuring equal equilibrium swelling states of all hydrogels as demonstrated by the kinetic curves presented in Figure S26. The ability of hydrogels to absorb water is usually described by the swelling ratio *Q*, which is given by eq 1 and defines the weight percentage of water in each sample.^67,68^ In this context, *M*_s_ denotes the weight of the fully swollen sample, whereas *M*_d_ expresses the weight of the dry state.
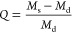
1As with the mechanical properties of poly(vinylphosphonate)-based hydrogels, a correlation was found between initial copolymer composition and water uptake of the cross-linked material (Figure 3).

**Scheme 3:**
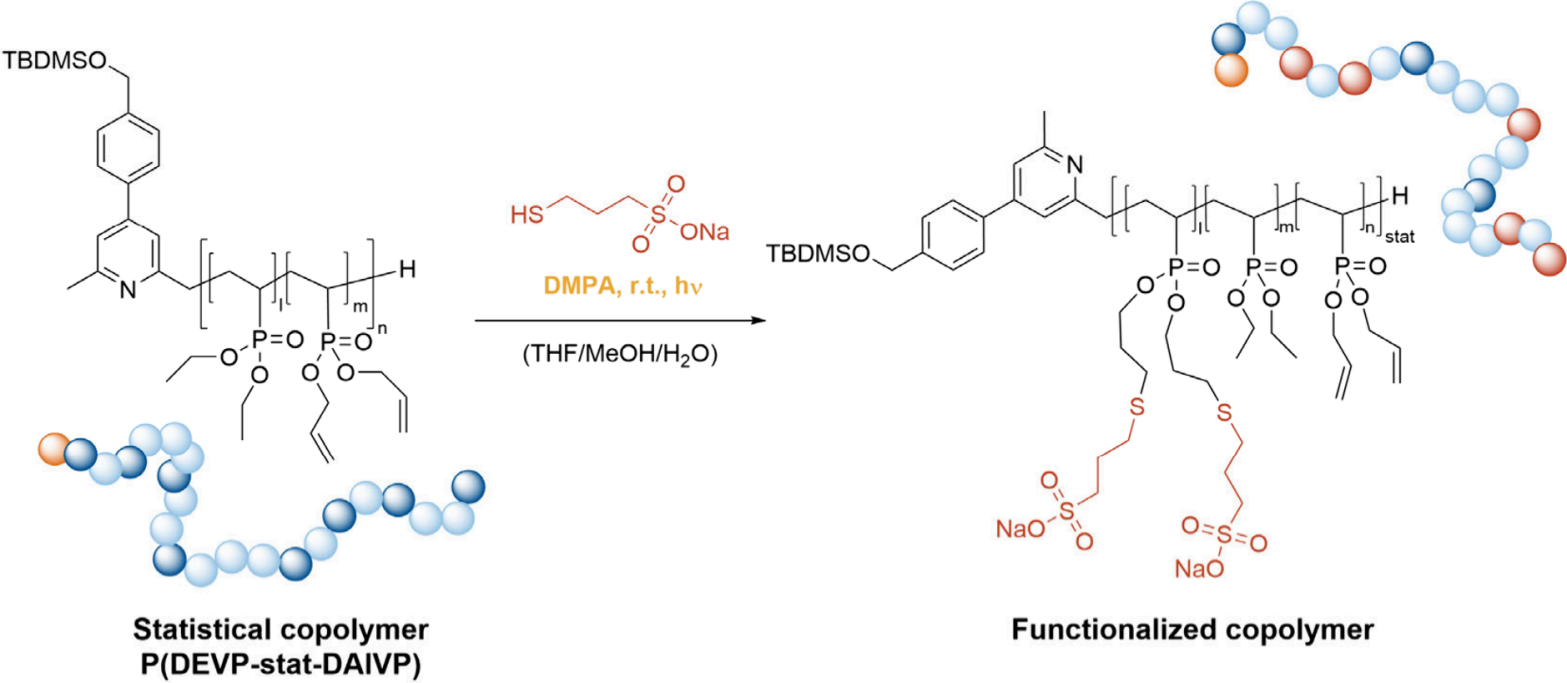
Functionalization of P(DEVP-stat-DAlVP) with Sodium 3-Mercaptopropane-1-sulfonate Prior to Hydrogel Synthesis through Photoinitiated Thiol–ene Click Chemistry

Unlike the mechanical properties, the swelling ratio decreases with an increasing number of DAlVP units and, therefore, cross-links in the material. This can be attributed to reduced chain mobility in more cross-linked networks hindering the solvation of the chains as well as the higher hydrophobicity of the DAlVP compared to the more hydrophilic DEVP monomer. The highest water absorption of 2.22 g of water per gram of dry hydrogel was achieved for a DAlVP content of 5.7%. Further, studying the hydrolytic stability of these novel materials under physiological conditions in phosphate-buffered saline solution (pH = 7.4) at 37 °C revealed no significant degradation over 8 days (Figure S27). To increase the water uptake, the copolymers (Table 1, entry 3 and entries 6–8) were functionalized by performing a click reaction with sodium 3-mercaptopropane-1-sulfonate prior to cross-linking as illustrated in Scheme 3.

The modified polymers were purified via dialysis against water (MWCO = 8 kDa), and successful functionalization was confirmed via ^1^H DOSY NMR spectroscopy (Figure S28). Unfortunately, a comparison of the ^1^H NMR spectra of each copolymer before and after functionalization with the sulfonate could not afford the degree of functionalization due to the lack of a reference signal in both spectra (Figure S29). However, ^1^H NMR spectroscopy gave access to the composition of unfunctionalized polymers, whereas elemental analysis of the functionalized polymers (Table S2) led to the compositions displayed in Table 2. Subsequently, the purified samples were successfully subjected to hydrogel formation applying thiol–ene click chemistry (for experimental details, see the Supporting Information). The resulting hydrogels were dried in vacuo, and their water uptake was investigated in the same manner as that described above. In this context, each hydrogel synthesis and water absorption experiment was performed in triplicate. The water uptake regarding the polymer composition and a description of the mechanical properties of the swollen hydrogels are provided in Table 2.

**Table 2:** Calculated Compositions of P(DEVP-*stat*-DAlVP) Copolymers Functionalized with Sodium 3-Mercaptopropane-1-sulfonate, Water Uptake of the Corresponding Hydrogels, and Description of the Mechanical Properties of the Water-Swollen Hydrogel Samples

entry	DEVP [%]^a^	DAlVP [%]^a^	functionalized [%]^a^	water uptake *Q* [g(H_2_O)/g(sample)]^b^	description of mechanical properties
9	96.2	3.11	0.69	50 ± 5	no structural integrity
10	90.1	7.82	2.08	54 ± 1	no structural integrity
11	84.7	11.4	3.90	39 ± 3	soft and brittle
12	79.4	16.5	4.10	15 ± 0	soft and brittle

a Polymer composition of functionalized samples after purification, as determined from ^1^H NMR spectroscopy and elemental analysis.

b Water uptake of corresponding hydrogels with standard deviation. *Q* = (*M*_s_ – *M*_d_)/*M*_d_.

As reflected by the compositions in Table 2, starting with P(DEVP-*stat*-DAlVP) copolymers with an increasing number of DAlVP units (Table 1, entries 3 and 6–8) results in different degrees of functionalization upon application of the thiol–ene reaction with sulfonate. In general, the values for the water uptake presented in Table 2 reveal an extraordinary increase in the water uptake of these novel materials. More specifically, hydrogels originating from the functionalized polymers exhibited a roughly 25-fold increase in water uptake up to more than 50 g of water per gram of material (Table 2) compared to the initially applied polymers (Figure 3). This renders these materials applicable as superabsorbers and can be attributed to a significant increase in the hydrophilicity of the polymers used in hydrogel synthesis. Upon introduction of the sulfonate side chains, ionic moieties are introduced into the hydrogel networks. Similarly to acrylic acid–based superabsorbers, introducing sodium salts of deprotonated acids causes a significant increase in water absorption due to the solvation of both anions and cations.^69^ When correlating the polymer composition to the water uptake, a significant increase in the water uptake at lower DAlVP contents is obtained, which, as explained above, is due to a lower cross-linking density. Notably, the amount of functionalized monomer units increased along with the DAlVP content from entries 9 to 12. Therefore, the hydrophilicity-enhancing effect of sulfonate side chains appears to be compensated by a higher number of cross-links in entry 12, for example. This is evidenced by analyzing entries 11 and 12, which exhibited similar amounts of sulfonate-functionalized monomers. Neglecting the differences in the DEVP amounts, the significantly reduced water uptake of hydrogels synthesized from polymer 12 can be attributed to the higher DAlVP ratio. Comparing entries 9 and 10, hydrogels corresponding to the presented terpolymer compositions revealed almost identical swelling ratios. This could potentially demonstrate the interplay between a decreasing number of cross-links (entry 9) and an increasing number of hydrophilic side groups (entry 10). The mechanical properties of the swollen hydrogels were dominated by the amount of DAlVP units available for cross-linking, as denoted in Table 2, which can also be seen in the images of the corresponding swollen networks presented in Figure S30. The correlation between the polymer composition and the mechanical properties of the respective hydrogel is in good accordance with the findings presented earlier in this work. The findings were further complemented by oscillatory frequency sweeps, revealing increasing storage moduli of dilute, fully cross-linked solutions of entries 9 to 12, aligning well with the increasing DAlVP content in these samples (Figure S31). To conclude, the combination of functionalizing P(DEVP-stat-DAlVP) via thiol–ene click chemistry, followed by cross-linking to hydrogels, demonstrated by this example, could be expanded toward introducing other functionalities into the final materials such as catalytic motifs or biologically active substrates.

### Hydrogel Purification and Biocompatibility Studies

**Figure 4:**
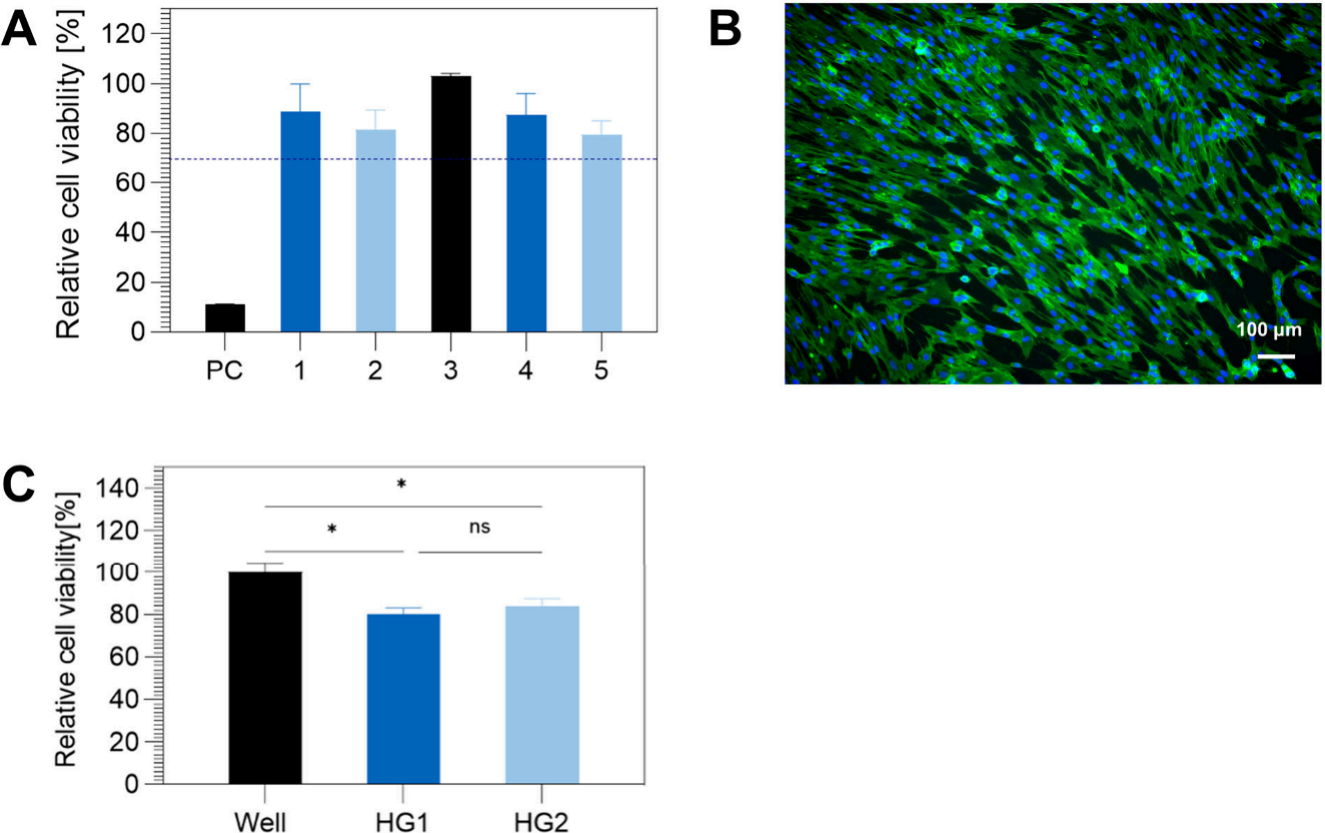
(A) Cytotoxicity test of human umbilical artery smooth muscle cells (HUASMCs) based on the extract test, according to ISO 10993. The cell viability is shown relative to the negative control (NC: untreated medium) upon incubation of the cells with the eluates of Hydrogels 1–5 (Table S3) at 37 °C for 72 h. The eluate of a latex glove served as a positive cytotoxic control (PC). Samples demonstrating cell viability higher than the threshold of 70% are considered noncytotoxic according to ISO 10993. (B) Cell adhesion of human umbilical artery smooth muscle cells (HUASMCs) on poly(vinylphosphonate)-based hydrogels after incubation for 3 days. Scale bar = 100 μm, nuclei are stained in blue and actin in green. (C) Cell viability of smooth muscle cells 3 days after seeding directly on two poly(vinylphosphonate)-based hydrogel samples (HG1 and HG2) relative to the viability on the well plate surface as a control.

Finally, cytotoxicity investigations were conducted to explore these novel materials’ potential in biomedical applications. Initial cytotoxicity tests based on the extract test performed according to ISO 10993 with the unpurified, dried samples revealed relatively low cell viabilities (40–60%) relative to the control (untreated medium) upon incubation of human umbilical artery smooth muscle cells (HUASMCs) with the eluates of the hydrogels at 37 °C for 72 h. Therefore, an additional purification step was introduced after the hydrogel synthesis. In this context, different hydrogel samples were subjected to Soxhlet extraction with either ethanol followed by water or water exclusively. In all cases, this resulted in a significant weight loss of the samples and hinted toward the leakage of potentially cytotoxic compounds as indicated by the ratios of extractable compounds and gel calculated for different hydrogels (Table S3). Further, a ^1^H NMR spectrum of the extractable fraction after solvent removal gave evidence of cross-linker, initiator, and its’ decomposition products, which were removed from the sample upon Soxhlet extraction (Figure S32). With the purified hydrogels, the cytotoxicity test based on the extract test described above was repeated. The purified hydrogel samples, in which we screened different copolymers, cross-linkers, and purification methods (Table S3) exhibited cell viabilities relative to the control above the 70% threshold defined by the ISO 10993, and were therefore declared noncytotoxic (Figures 4A and S33). Finally, the purified hydrogels were tested for cell adhesion to be evaluated as potential scaffold materials for tissue engineering. Figure 4B shows the cell adhesion to and distribution on the hydrogel surface after incubation with smooth muscle cells for 3 days. Further, an alignment of smooth muscle cells could be detected, which could not be explained through any aspects of sample preparation and is part of ongoing research. Figure 4C shows the results of the cell adhesion test and demonstrates the cell viability of smooth muscle cells 3 days after seeding directly on the poly(vinylphosphonate)-based hydrogels relative to that on the noncytotoxic surface of the well plate (control surface). Considering this data, it becomes evident that these novel, cytocompatible materials can already be applied as scaffold materials for HUASMCs and be further investigated based on their biological and mechanical properties for their potential in biomedical applications such as tissue engineering. An investigation of the endothelialization of the hydrogels was conducted with human umbilical vein endothelial cells seeded on the surfaces of hydrogels HG1 and HG2. After a 24 h incubation step, the cells were stained, and the layer formation was examined. Images show that both hydrogels supported the adhesion, growth and layer formation of endothelial cells, as shown by CD31 expression in Figure S34. These results show the broad potential of the hydrogels as they could support the endothelialization of tissue-engineered cardiovascular constructs and thereby reduce the risk of thrombosis and inflammation.

**Figure 5:**
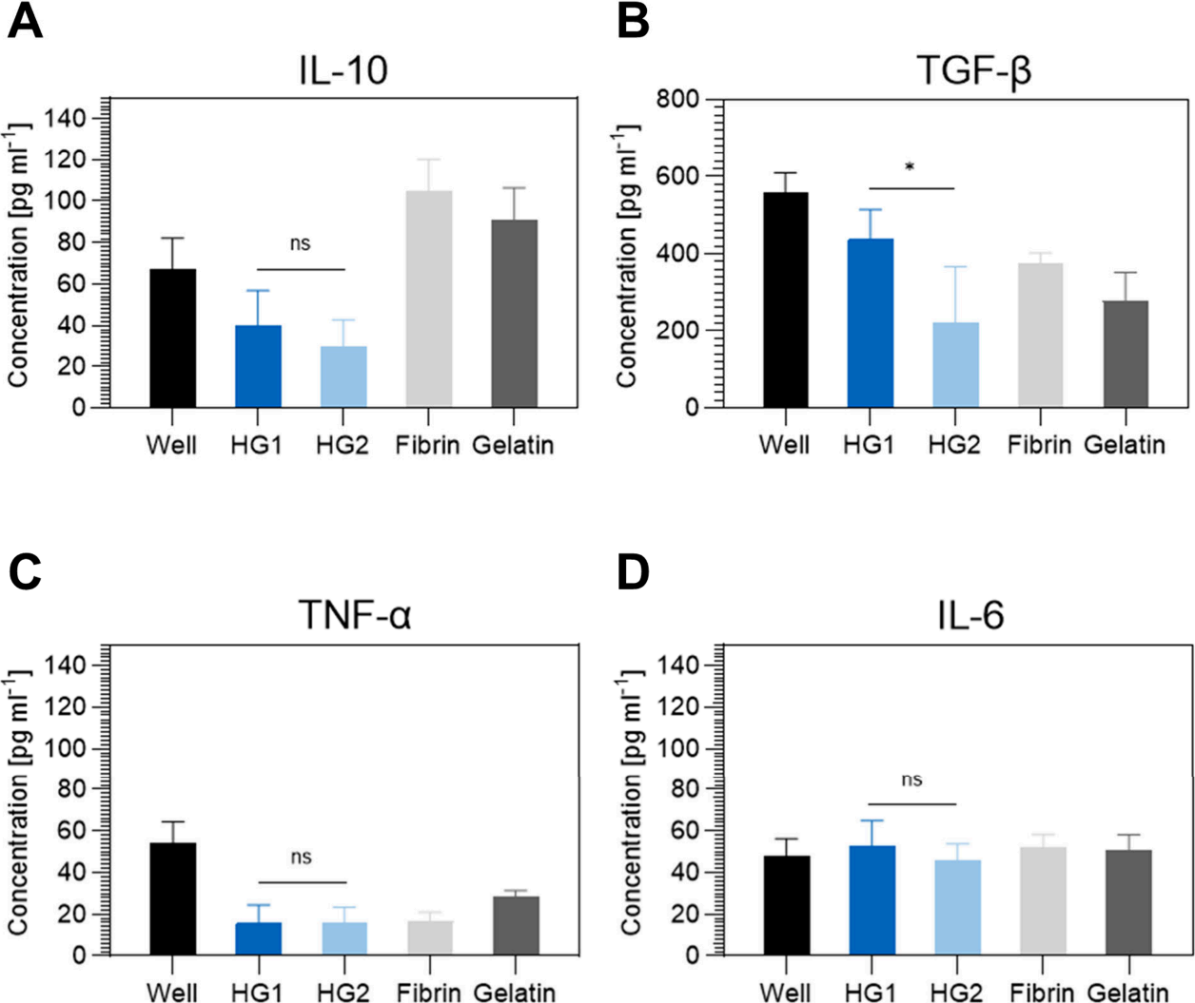
Cytokine expression of THP-1 monocyte-derived M0 macrophages after cultivation on the hydrogel samples (HG1, HG2), tissue culture polystyrene (Well), fibrin gel, and gelatin gel. Concentrations of anti-inflammatory cytokines interleukin-10 (IL-10) (A) and transforming growth factor-beta (TGF-β) (B) and pro-inflammatory cytokines tumor necrosis factor-alpha (TNF-α) (C) and interleukin-6 (IL-6) (D) after a 72 h incubation. Values represent mean ± standard deviation obtained from 5 independent samples. An unpaired *t* test was conducted to compare the two sample groups.

Additionally, the host immune response toward these novel materials was tested and compared to the benchmark systems fibrin and gelatin to further elucidate the potential of applying these scaffolds in tissue engineering. In this context, inflammation studies with purified P(DEVP-*stat*-DAlVP)-based hydrogels were conducted in vitro by measuring the cytokine release of THP-1 monocyte-derived M0 macrophages seeded onto the hydrogels. The hydrogels denoted as HG1 and HG2 consisted of cross-linked polymers with 15% and 20% DAlVP units, respectively. To quantify the immune response, the release of the anti-inflammatory cytokines interleukin-10 (IL-10) and transforming growth factor-beta (TGF-β) and the pro-inflammatory cytokines tumor necrosis factor-alpha (TNF-α) and interleukin-6 (IL-6) were quantified. As shown in Figure 5, the released cytokine concentrations of IL-6, TNF-α, and the anti-inflammatory cytokine IL-10 show no significant difference for the two hydrogel samples. Regarding the release of TGF-β, HG1 exhibits higher values than HG2, potentially attributed to differences in the composition (monomer ratios, different amounts of incorporated PEG-based cross-linker, etc.). In general, however, the results of these experiments indicate that the novel poly(vinylphosphonate)-based hydrogels perform similarly to hydrogels commonly applied in tissue engineering applications, underlining their great potential for biomedical applications.

**Figure 6:**
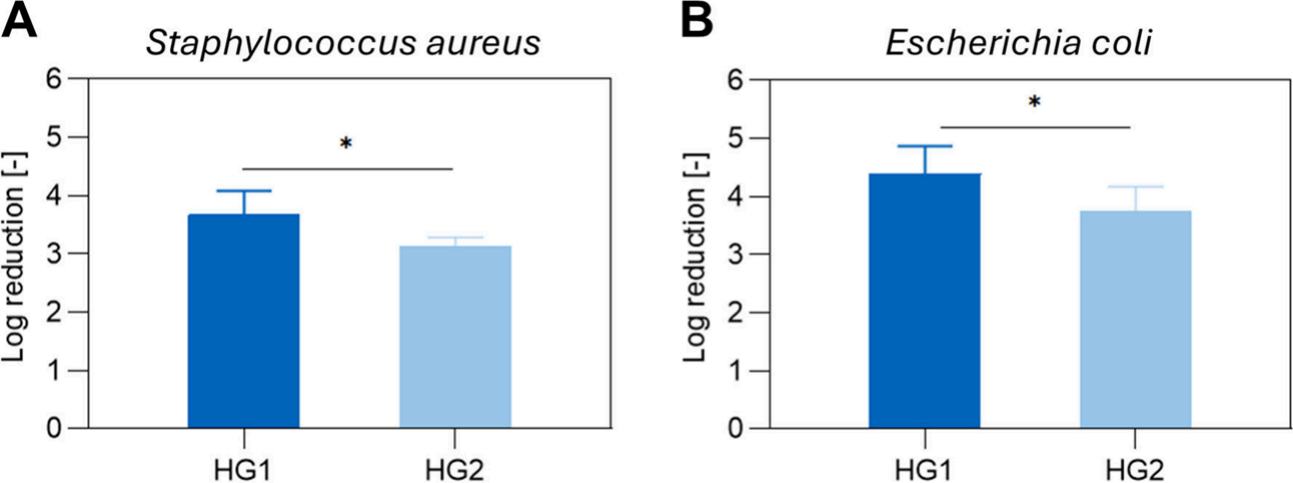
*S. aureus* (A) and *E. coli* (B) adhesion to P(DEVP-*stat*-DAlVP)-based hydrogels. The reduction values represent mean ± standard deviation obtained from 5 independent samples. An unpaired *t* test was conducted to compare the two sample groups.

Lastly, the antibacterial properties of HG1 and HG2 were explored by performing bacterial adhesion tests with *Staphylococcus aureus* and *Escherichia coli* after seeding directly onto the hydrogel surfaces and incubation at 37 °C for three hours. Quantification of the adherent bacteria on the samples yielded log reduction values between 3 and 4 for both bacterial strains, corresponding to a 99.9–99.99% reduction of bacteria (Figure 6), which indicates the antibacterial properties of these hydrogels.

## Conclusion

To summarize, this study successfully demonstrated the synthesis of hydrogels from statistical P(DEVP-*stat*-DAlVP) copolymers upon application of photoinitiated thiol–ene click chemistry. The copolymerization allows a facile adjustment of chain length and copolymer composition through variation of the monomer feed and monomer/catalyst ratio, while maintaining narrow polydispersities. After a series of preliminary experiments, the cross-linking process could be visualized by time-resolved rheological experiments with coupling to a UV lamp. Subsequently, oscillatory rheology was applied to characterize the mechanical properties of the various hydrogels. In this context, the mechanical strength of cross-linked samples increased with higher amounts of allyl group-containing DAlVP units in the copolymer as well as upon application of linkers containing more than two thiol functionalities. Mechanical characterization of different cross-linked samples by nanoindentation revealed increasing sample hardness and stiffness with higher cross-linking degrees and a significantly reduced hardness in the water-swollen state of the gels. Regarding the water uptake, more cross-linked hydrogels exhibited lower water absorption, attributed to decreased chain mobility and overall increased hydrophobicity of the samples. However, a significant increase in the swelling ratio was observed with the utilization of sulfonate-functionalized polymers for hydrogel synthesis. Despite showing a 25-fold increase in the water absorption capacity, which was assigned to the increased hydrophilicity, the water uptake was seemingly still governed by the cross-linking density. Finally, the hydrogels exhibited cytocompatibility after a successful purification via Soxhlet extraction. Further, smooth muscle cells readily proliferated and adhered on the surfaces of purified poly(vinylphosphonate)-based hydrogels 3 days after direct seeding. Additional biocompatibility investigations revealed the ability to support endothelialization, no pro-inflammatory response toward these novel hydrogels, and antibacterial properties based on a reduction of the adhesion of *S. aureus* and *E. coli* on the sample surfaces. Overall, these widely tunable material properties in terms of mechanical performance and swelling behavior, in combination with low cytotoxicity, render these hydrogels appealing candidates for potential biomedical applications such as tissue engineering. Additionally, the fundamental structure–property relationships of polymer to hydrogel presented in this study offer a platform for further research ambitions. In this context, a profound understanding of the material properties of poly(vinylphosphonate)-based hydrogels can contribute to ongoing studies on the application of this photochemical cross-linking process in, e.g., additive manufacturing of these novel materials.

## Supporting Information Available

Materials and methods; Polymer characterization; Experimental procedures; Hydrogel synthesis and characterization; Rheological control experiments; Swelling kinetics; Elemental analysis of functionalized copolymers; Hydrogel purification; Cytotoxicity testing; Additional data for biological tests (PDF)

## Abbreviations

DAlVP: diallyl vinylphosphonate
DEVP: diethyl vinylphosphonate
REM-GTP: rare earth metal-mediated group-transfer polymerization
HUASMCs: human umbilical artery smooth muscle cells
P(DEVP-*stat*-DAlVP): statistical copolymers composed of DEVP and DAlVP
LVR: linear viscoelastic region
DMPA: 2,2-dimethoxy-2-phenylacetophenone
AIBN: azobisisobutyronitrile
IE: initiator efficiency
*Đ*: polydispersity index
PEG: polyethylene glycol.
