# Supplementary material for: Cytocompatible Hydrogels with Tunable Mechanical Strength and Adjustable Swelling Properties through Photo-Cross-Linking of Poly(vinylphosphonates)
Source: ACS Appl Mater Interfaces. 2024 Oct 15;16(43):58135–47. doi: 10.1021/acsami.4c07860 (PMC11533175; doi:10.1021/acsami.4c07860)
Supplement: Supplementary file 1 — am4c07860_si_001.pdf [file am4c07860_si_001.pdf]

Supporting Information

Cytocompatible Hydrogels with Tunable Mechanical Strength and Adjustable Swelling Properties through Photo-Cross-Linking of Poly(vinylphosphonates)

Anton S. Maier,[†] *Salma Mansi,*[‡] *Kerstin Halama,*[†] *Philipp Weingarten,*[†] *Petra Mela,*[‡] *and Bernhard Rieger*[*,†]

[†] Technical University of Munich, Germany, TUM School of Natural Sciences, Department of Chemistry, WACKER-Chair of Macromolecular Chemistry, Lichtenbergstraße 4, 85748 Garching, Germany

[‡] Technical University of Munich, Germany, TUM School of Engineering and Design, Department of Mechanical Engineering, Chair of Medical Materials and Implants, Munich Institute of Biomedical Engineering, Munich Institute of Integrated Materials, Energy and Process Engineering, Boltzmannstraße 15, 85748 Garching, Germany

Corresponding Authors

* rieger@tum.de

TABLE OF CONTENTS

1. Materials and Methods 3
2. Statistical Copolymerization of Diethylvinylphosphonate (DEVP) and Diallylvinylphosphonate (DAlVP) 8
3. Hydrogel Synthesis and Characterization 18
4. Hydrogel Purification and Cytotoxicity Testing 34
5. Additional biocompatibility studies 39
6. References 42

1. MATERIALS AND METHODS

General Experimental

All air and moisture-sensitive compounds were prepared using standard Schlenk techniques or in a glovebox with argon (99.996 vol.-%) from Westfalen as an inert gas. All glass instruments were oven-dried prior to use. Unless otherwise stated, all chemicals and solvents were purchased from Sigma-Aldrich, ABCR GmbH, or TCI Chemicals and used without further purification. Dry solvents were obtained from an MBraun MB-SPS-800 solvent purification system or by drying over activated alumina and stored over activated 3 Å molecular sieves. Deuterated solvents were purchased from Sigma-Aldrich and dried over activated 3 Å molecular sieves. The monomers diethyl vinylphosphonate (DEVP), diallyl vinylphosphonate (DAlVP), the complex $Cp_2YCH_2TMS(thf)$ and the initiator 4-(4-(((tert-butyldimethyl-silyl)oxy)methyl)phenyl)-2,6-dimethylpyridine were synthesized according to literature-known procedures.[1–3] The monomers were dried over calcium hydride and distilled prior to polymerization.

Elemental analysis

All elemental analyses were performed by the Laboratory for Microanalysis at the Institute of Inorganic Chemistry at the Technical University of Munich.

LCST determination

The LCST was determined via DLS analysis on a Litesizer 500 particle size analyzer equipped with a 658 nm 40 mW laser diode. The samples were transferred into a 45 µL low volume quartz cuvette (3x3 mm light path, Hellma Analytics). The lower critical solution temperature was determined by measuring the transmittance of the sample between 20°C and 60 °C over one heating and one cooling cycle. Each temperature was equilibrated for 4 minutes, and the

transmittance was measured over 6 seconds. The sample concentration of polymer was 2 mg mL^{-1} in distilled water, and the sample was passed through a 0.45 µm syringe filter before measuring. The cloud point was determined at a 10% decrease of the transmittance compared to the initial value.

Thermogravimetric analysis (TGA)

Thermogravimetric analyses were recorded on a Q5000 SA from TA Instruments. The samples were applied in tared platinum crucibles and heated from room temperature to 800 or 1000 °C under synthetic air, applying a continuous heating ramp of 10 K/min. The measurement data were analyzed using the TA Universal Analysis software.

Differential scanning calorimetry (DSC)

DSC measurements were recorded on a DSC Q2000 from TA Instruments in exo down mode. The temperature program consisted of three consecutive heating and cooling cycles with a continuous heating ramp of 5 K min^{-1} between -70 °C and 170 °C. The measurement data was analyzed using the TA Universal Analysis software.

Infrared spectroscopy (FT-IR)

The IR-spectra were recorded on a Vertex-70 FT-IR spectrometer from Bruker at room temperature.

Oscillatory rheology

Rheological characterizations were performed on an MCR 302 controlled-stress rheometer from Anton Paar with an upper plate (25 mm diameter) and a glass plate as counterpart (gap size 0.5 mm). The samples were applied in a liquid state (250 µL) and tempered to 25 °C through an

upper and lower Peltier system. Additionally, a protective hood was used. To prevent solvent evaporation during the measurements, a circular, moisturized sponge was placed around the sample under the protective hood while avoiding contact with the sample. A MAX-302 lamp from Asahi Spectra with a cutoff wavelength below 400 nm was used for irradiation through the bottom glass plate. Measurements were performed every 10 seconds, and the data was monitored via the Rheoplus software. Additional parameters are specified in the description of the corresponding measurement result.

Nanoindentation

To measure the surface mechanical properties of the crosslinked samples, nanoindentation was performed in air and after swelling in distilled water with a Bioindenter (UNHT^3) from Anton Paar (Graz, Austria) with a ruby spherical indenter of 500 µm radius. The loading rate was set from 40 µN min^{-1} to a maximum of 20 µN min^{-1} with a 30-second hold at the maximum load followed by unloading at 40 µN min^{-1}. The maximum indentation depth was fixed to 100 µm. For the experiments in water, the specimen was fixed on the Petri dish surface using duct tape and swollen for 120 minutes. The indentation modulus was calculated using a Hertzian fit to the loading data. For each sample, 6 measurements were performed with a sideward displacement of 500 µm.

Photochemical reactions

To induce photochemical crosslinking, the samples were irradiated at a 20 mm distance, resulting in a light power intensity of 150 mW cm^{-2}, applying a current of 700 mA and a forward voltage of 15.2 V.

Lyophilization

Lyophilization was performed on a VaCo 5-II-D from Zirbus Technology GmbH with a pressure of 2 mbar and a condenser temperature of -90 °C. Polymers subjected to freeze-drying were dissolved in 1,4-dioxane or distilled water before freezing in liquid nitrogen under constant rotation.

Size-exclusion chromatography multi-angle light scattering (SEC-MALS)

Polydispersities of the polymers were determined via size-exclusion chromatography (SEC) with sample concentrations of 4 mg mL^{-1} on two PL Polargel-M columns (Agilent) at 40 °C. A mixture of water and THF (1:1), treated with tetrabutylammonium bromide (9 g L^{-1}) and 3,5-di-tert-butyl-4-hydroxytoluene (340 mg L^{-1}) was used as eluent. Samples were analyzed using a Wyatt Dawn Heleos II light scattering unit in combination with a Wyatt Optilab rEX as RI detector unit.

Nuclear magnetic resonance spectroscopy (NMR)

^{1}H-NMR- and ^{31}P-NMR spectra of polymers were recorded on a Bruker AV-400HD, AV-500HD, or AV-II-500 spectrometer at 400 or 500 MHz (^{1}H) and 203 MHz (^{31}P), respectively. All chemical shifts are given in parts per million (ppm) and referenced to the residual proton signal of the respective solvent (Benzene-d6: δ = 7.16 ppm, Methanol-d4: δ =3.31 ppm). Deuterated solvents were purchased from Sigma-Aldrich or Deutero and dried over activated 3 Å molecular sieves. The NMR spectra were analyzed using the MestReNova software. ^{1}H DOSY NMR experiments were performed to characterize copolymers and functionalized copolymers.

Statistical analysis

The results of the cytotoxicity tests are expressed as the mean ± standard deviation. The Shapiro-Wilk test was used to test normal distribution. When homogeneity of variances was fulfilled, the unpaired two-sided t-test between the groups with normal distribution was conducted. The unpaired two-sided t-test with Welch's correction was conducted for samples with unequal variances. The Mann-Whitney test was applied for samples without normal distribution. A statistically significant level was determined at $p < 0.05$. Data were analyzed using GraphPad Prism 10.

2. STATISTICAL COPOLYMERIZATION OF DIETHYLVINYLPHOSPHONATE (DEVP) AND DIALLYLVINYLPHOSPHONATE (DALVP)

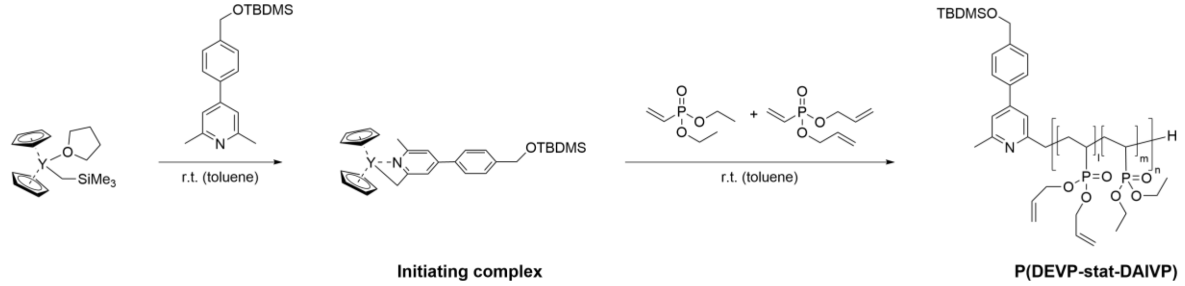

(1)

The statistical copolymerization of DEVP and DAlVP was performed according to literature-known procedures.[1,4] In a regular copolymerization experiment, the calculated amount of the catalyst precursor $Cp_2YCH_2TMS(thf)$ is dissolved in dry toluene, and the corresponding amount of 4-(4-(((tert-butyldimethyl-silyl)oxy)methyl)phenyl)-2,6-dimethylpyridine (1.10 eq.) added resulting in a yellow coloration of the solution upon formation of the initiating complex. The solution is stirred for two hours at room temperature before an aliquot (0.1 mL of the reaction mixture + 0.4 mL of dry benzene-d6) is removed from the reaction mixture to ensure quantitative conversion of the catalyst precursor via ^{1}H-NMR spectroscopy. To initiate polymerization, the monomers DEVP and DAlVP are weighed into a syringe, mixed, and added to the reaction mixture in one motion. Upon confirmation of quantitative conversion via ^{31}P-NMR spectroscopy by withdrawing another aliquot (0.1 mL of the reaction mixture + 0.4 mL of undried methanol-d4) from the reaction mixture, the polymerization is quenched by the addition of 0.5 mL of undried methanol. Subsequently, the polymer is precipitated from pentane. After centrifugation and decanting of the supernatant, the residual polymer was dissolved in 1,4-dioxane and subjected to freeze-drying, yielding the purified polymer.

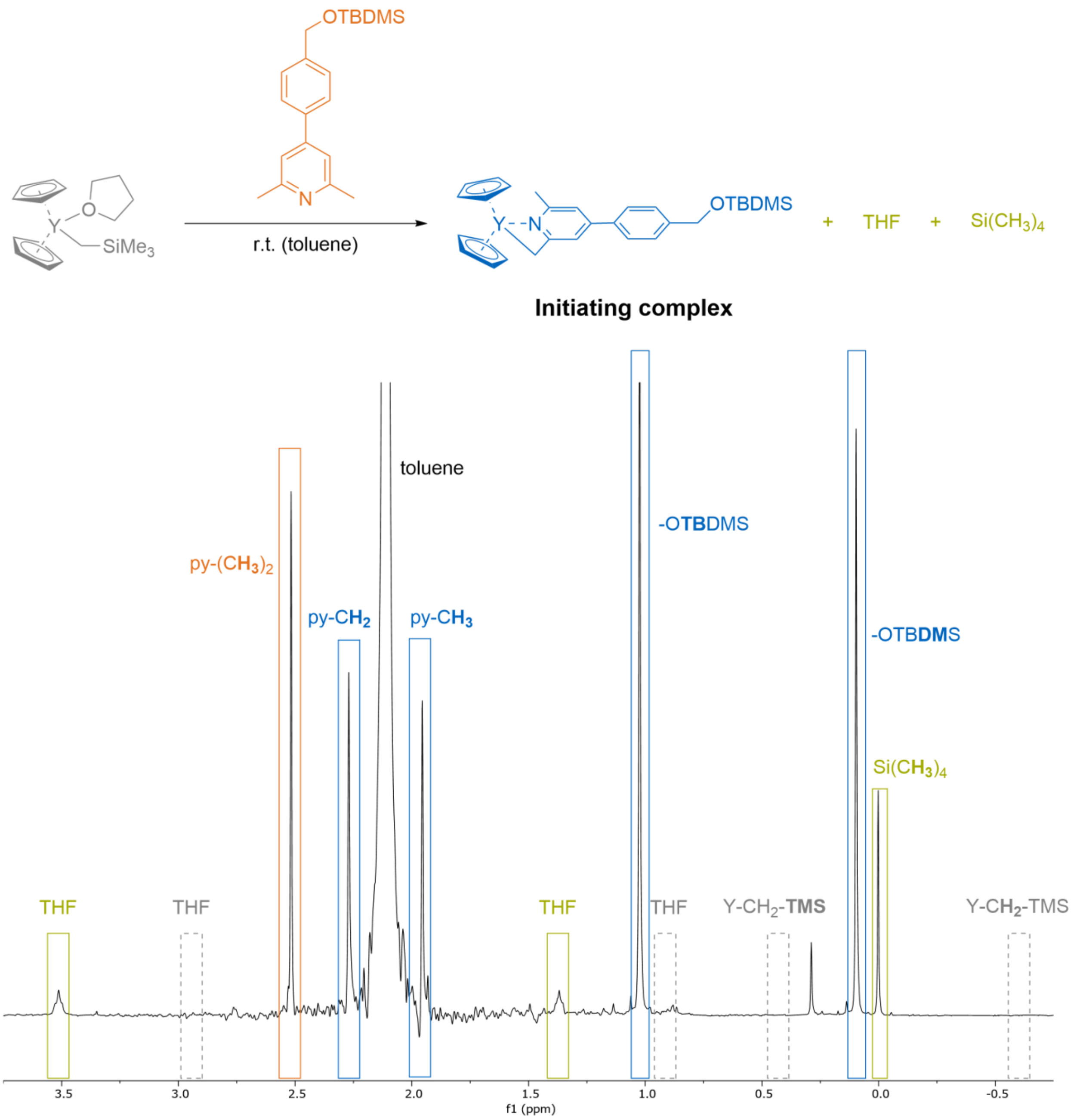

Figure S1: CH-bond activation of the initiator 4-(4-(((tert-butyldimethyl-silyl)oxy)methyl)phenyl)-2,6-dimethylpyridine (orange) with $Cp_2YCH_2TMS(thf)$ (grey) to yield the initiating complex (blue) and side products (green) and extract of the corresponding ^{1}H-NMR spectrum (Table 1, Entry 1). Dashed lines: catalyst signals prior to σ-bond metathesis.

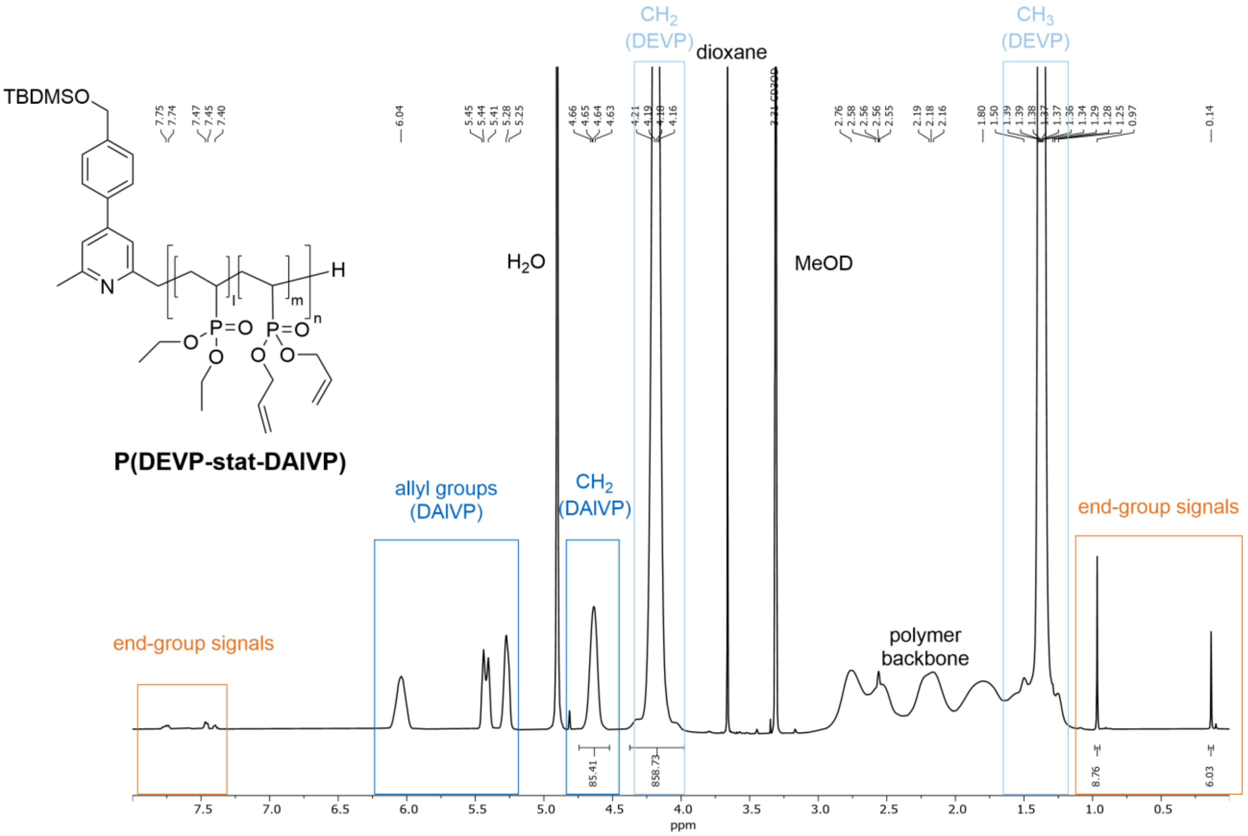

Figure S2: Exemplary copolymer ^{1}H-NMR spectrum of P(DEVP-stat-DAlVP) (Table 1, Entry 1) in MeOD with assignment of signals with relevant integrals for the determination of the molecular weight via ^{1}H-NMR spectroscopy.

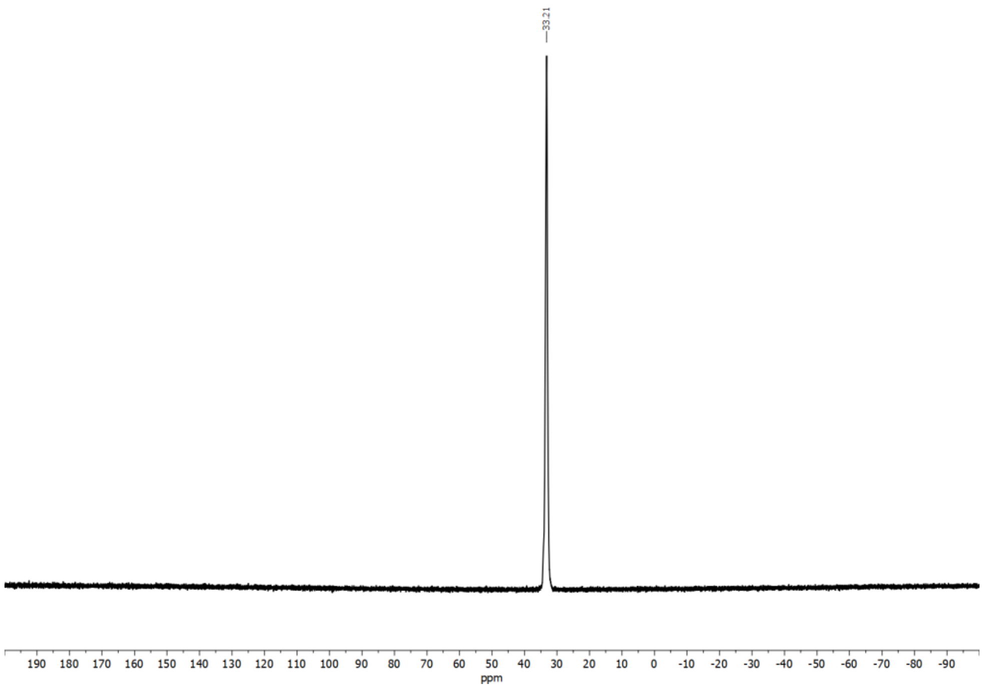

Figure S3: Exemplary ^{31}P-NMR spectrum of P(DEVP-stat-DAlVP) (Table 1, Entry 1) in MeOD.

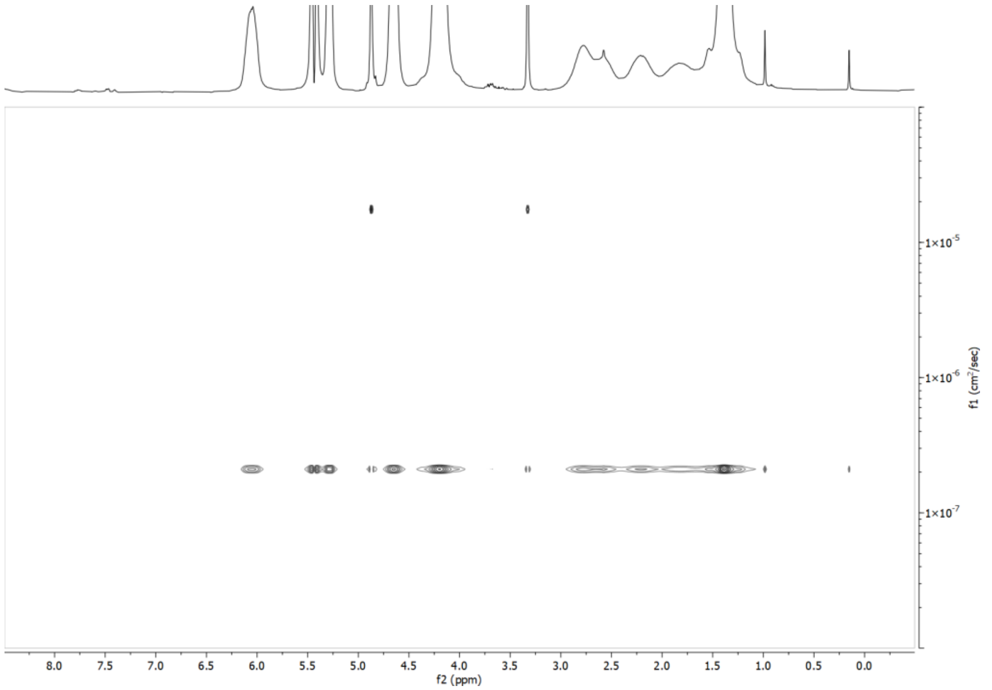

Figure S4: Exemplary ^{1}H DOSY NMR spectrum of P(DEVP-stat-DAlVP) (Table 1, Entry 6) in MeOD.

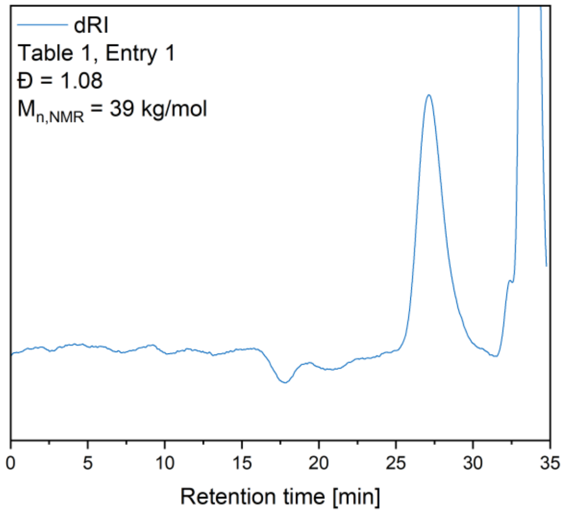

Figure S5: Refractive index detector signal of P(DEVP-stat-DAlVP) (Table 1, Entry 1) determined via SEC-MALS in THF/water (1:1) with tetrabutylammonium bromide (9 g L^{-1}) and 3,5-di-tert-butyl-4-hydroxytoluene (340 mg L^{-1}).

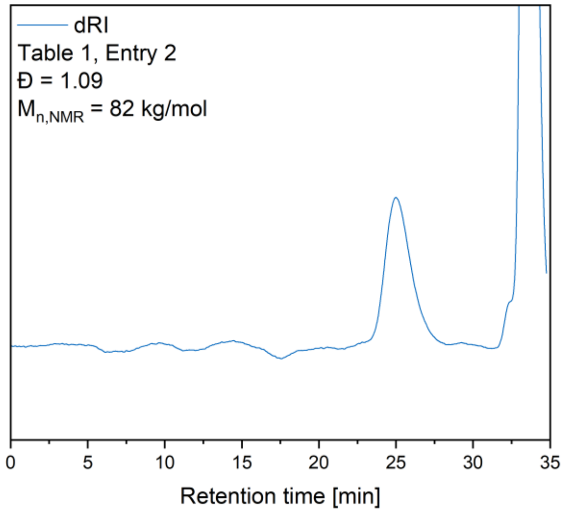

Figure S6: Refractive index detector signal of P(DEVP-stat-DAlVP) (Table 1, Entry 2) determined via SEC-MALS in THF/water (1:1) with tetrabutylammonium bromide (9 g L^{-1}) and 3,5-di-tert-butyl-4-hydroxytoluene (340 mg L^{-1}).

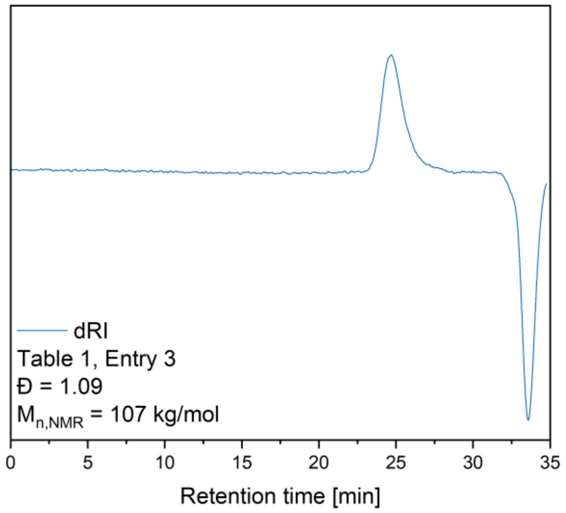

Figure S7: Refractive index detector signal of P(DEVP-stat-DAlVP) (Table 1, Entry 3) determined via SEC-MALS in THF/water (1:1) with tetrabutylammonium bromide (9 g L^{-1}) and 3,5-di-tert-butyl-4-hydroxytoluene (340 mg L^{-1}).

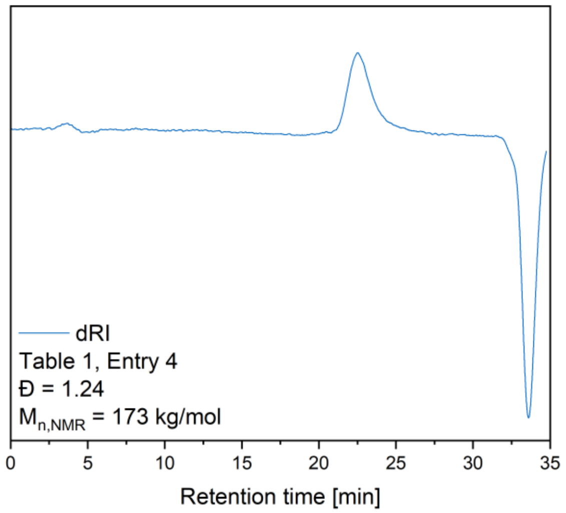

Figure S8: Refractive index detector signal of P(DEVP-stat-DAlVP) (Table 1, Entry 4) determined via SEC-MALS in THF/water (1:1) with tetrabutylammonium bromide (9 g L^{-1}) and 3,5-di-tert-butyl-4-hydroxytoluene (340 mg L^{-1}).

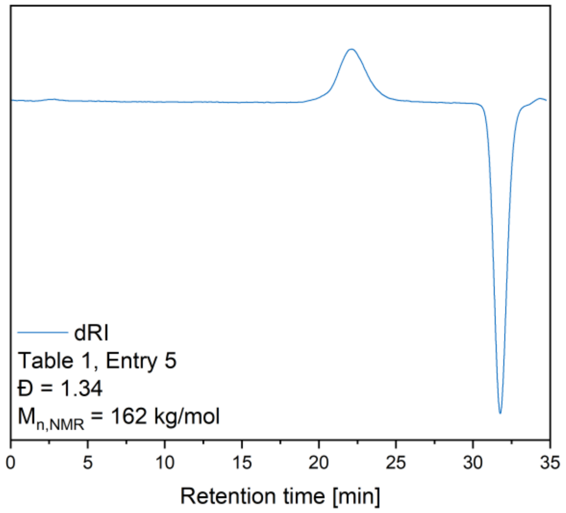

Figure S9: Refractive index detector signal of P(DEVP-stat-DAlVP) (Table 1, Entry 5) determined via SEC-MALS in THF/water (1:1) with tetrabutylammonium bromide (9 g L^{-1}) and 3,5-di-tert-butyl-4-hydroxytoluene (340 mg L^{-1}).

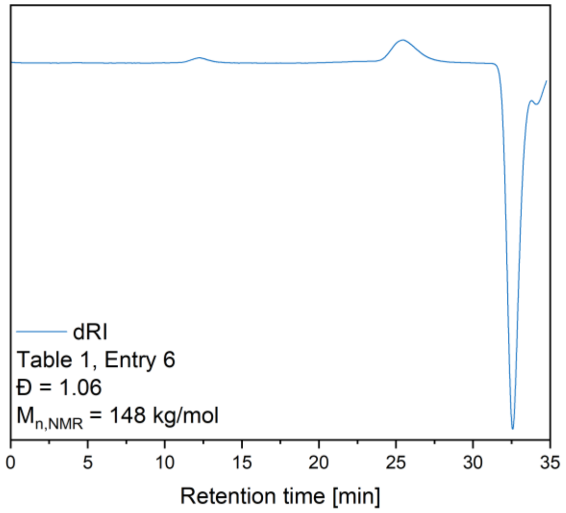

Figure S10: Refractive index detector signal of P(DEVP-stat-DAlVP) (Table 1, Entry 6) determined via SEC-MALS in THF/water (1:1) with tetrabutylammonium bromide (9 g L^{-1}) and 3,5-di-tert-butyl-4-hydroxytoluene (340 mg L^{-1}).

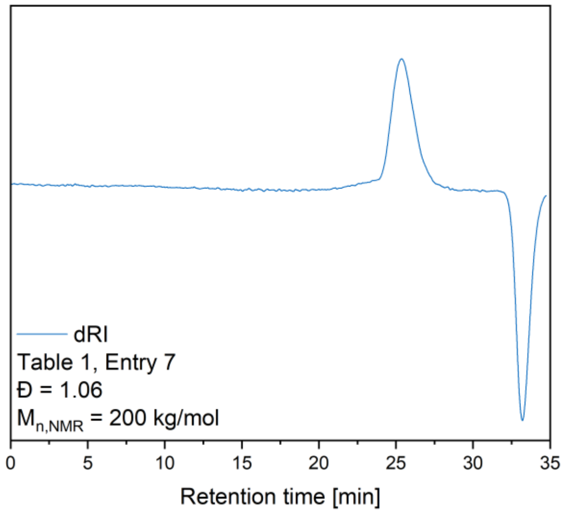

Figure S11: Refractive index detector signal of P(DEVP-stat-DAlVP) (Table 1, Entry 7) determined via SEC-MALS in THF/water (1:1) with tetrabutylammonium bromide (9 g L^{-1}) and 3,5-di-tert-butyl-4-hydroxytoluene (340 mg L^{-1}).

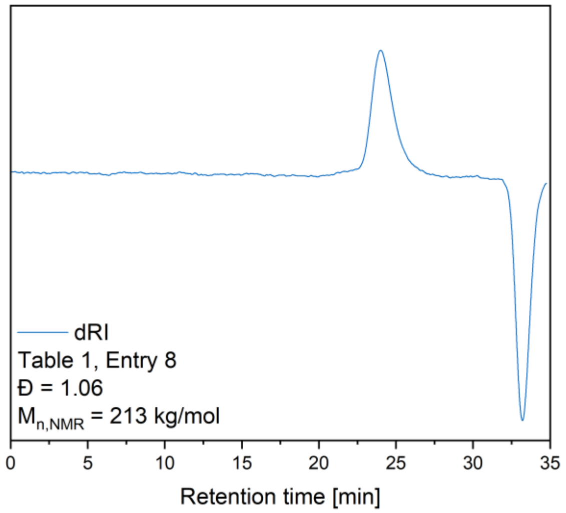

Figure S12: Refractive index detector signal of P(DEVP-stat-DAlVP) (Table 1, Entry 8) determined via SEC-MALS in THF/water (1:1) with tetrabutylammonium bromide (9 g L^{-1}) and 3,5-di-tert-butyl-4-hydroxytoluene (340 mg L^{-1}).

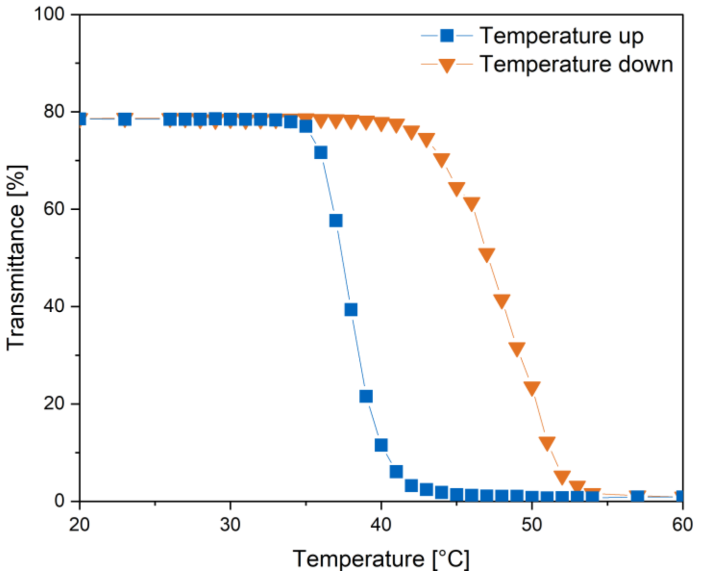

Figure S13: Determination of the cloud point of an aqueous solution of Entry 1, Table 1 via measurement of the transmittance of a 2 mg mL^{-1} solution of the polymer with increasing (blue symbols) and decreasing (orange symbols) temperature.

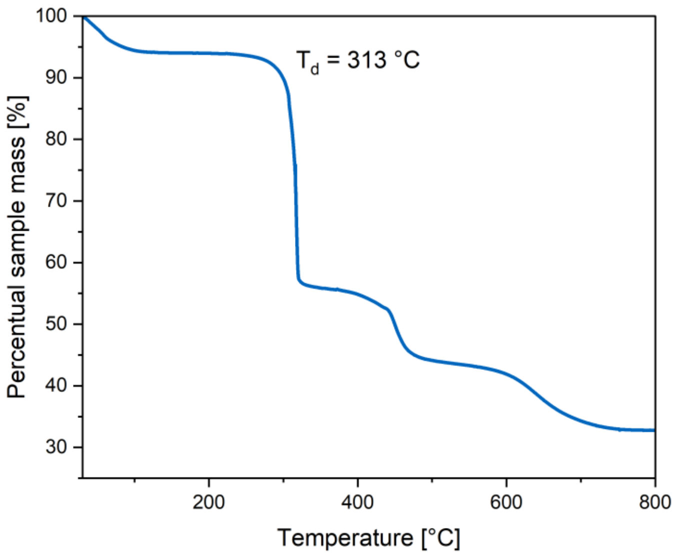

Figure S14: TGA measurement of Entry 3, Table 1 from room temperature to 800 °C under synthetic air with a heating rate of 10 K min^{-1}.

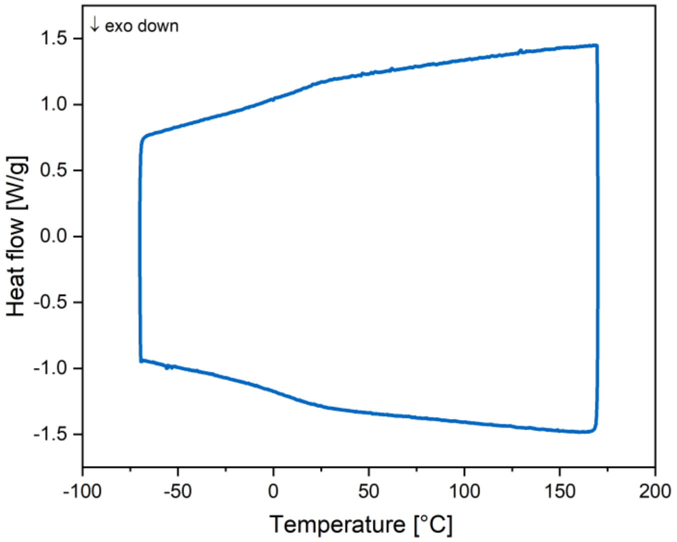

Figure S15: DSC measurement of Entry 3, Table 1 in the range of -70 to 170 °C measured in exo down mode.

3. HYDROGEL SYNTHESIS AND CHARACTERIZATION

Screening for hydrogel formation

Table S1: Screening of different reaction conditions and curing procedures for the formation of hydrogels from P(DEVP-stat-DAlVP) applying thiol-ene click chemistry with 3,6-dioxa-1,8-octanedithiol.

Entry	Reaction time	Curing procedure	Solvent	c (polymer) [mg/mL]	Degassed?	Gelation?
1	23 h	irradiation	THF/MeOH (5/1)	10	yes	yes
2	30 min	irradiation	THF/MeOH (5/1)	33	yes	yes
3	120 min	irradiation	**H_2O**	70	**no**	yes
4	120 min	irradiation	**1,4-dioxane**	160	**no**	yes
5	40 min	irradiation	1,4-dioxane	160	yes	yes
6	30 min	irradiation	1,4-dioxane	**666**	no	yes
7	60 min	**heating (AIBN)**	1,4-dioxane	160	no	yes

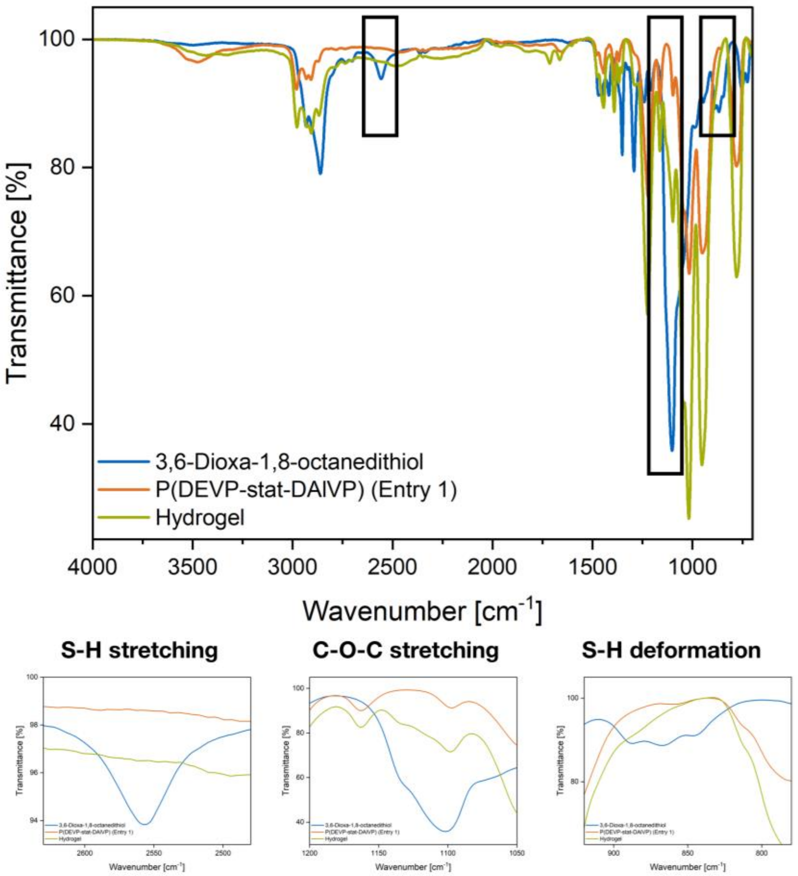

Figure S16: FT-IR spectra of the crosslinker (blue), the polymer (orange, Entry 1, Table 1), and the hydrogel (green): full spectra (top) and magnifications of the relevant regions in the full spectrum with assignments of signals (bottom).

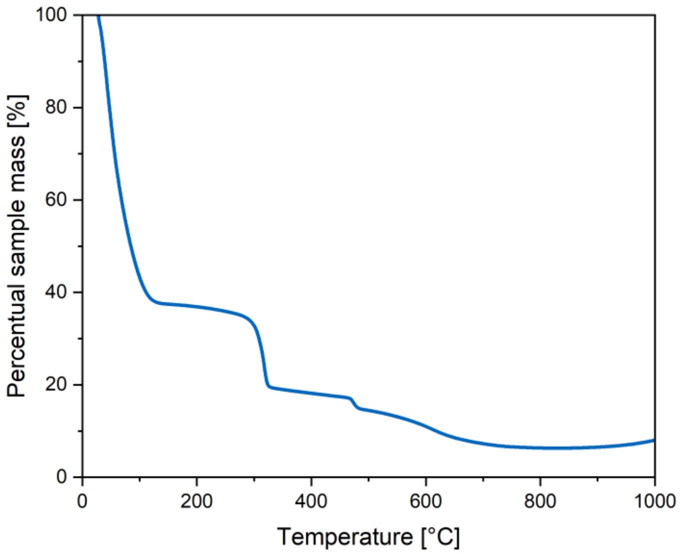

Figure S17: TGA measurement of a water-swollen hydrogel synthesized from Entry 8, Table 1 from room temperature to 1000 °C under synthetic air with a heating rate of 10 K min^{-1}.

Hydrogel synthesis for rheological experiments

In the rheological experiments described in the manuscript, gel samples subjected to characterization via oscillatory rheology were formed between the rheometer plates in situ. In a typical experiment, 100 mg of P(DEVP-stat-DAlVP) were dissolved in 0.3 mL of dioxane using a vortex mixer. Subsequently, the calculated amounts of 3,6-dioxa-1,8-octanedithiol (2.50 eq. with respect to the allyl groups of the polymer) and 2,2-dimethoxy-2-phenylacetophenone (0.40 eq. with respect to the allyl groups of the polymer) were added under light exclusion. Then, 0.25 mL of this stock solution was transferred onto the glass bottom plate of the rheometer, and the top plate was lowered. Next, the measurement was started, and irradiation of the sample from below (λ = 365 nm) was initiated at the desired time. In most cases, the sample behavior was studied for 5 minutes in the dark before measuring the rheological behavior upon irradiation for 5 minutes. As denoted in the manuscript, amplitude and frequency sweeps (Figures S13 and S14) on fully gelled samples to determine the linear viscoelastic region (LVR) were performed after irradiation of the polymer solutions between the plates for 5 minutes.

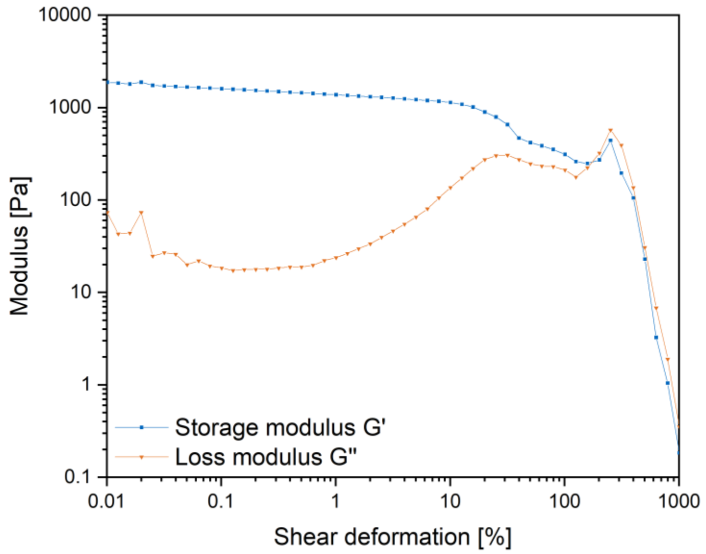

Figure S18: Amplitude sweep on a fully gelled sample with a frequency of 1 Hz and deformations between 0.01 and 1000%.

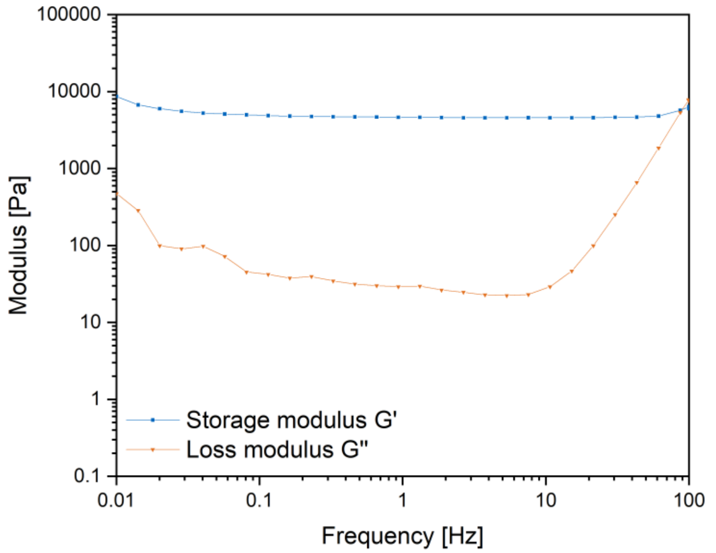

Figure S19: Frequency sweep on a fully gelled sample with a deformation of $\gamma = 1\%$ and frequencies between 0.01 and 100 Hz.

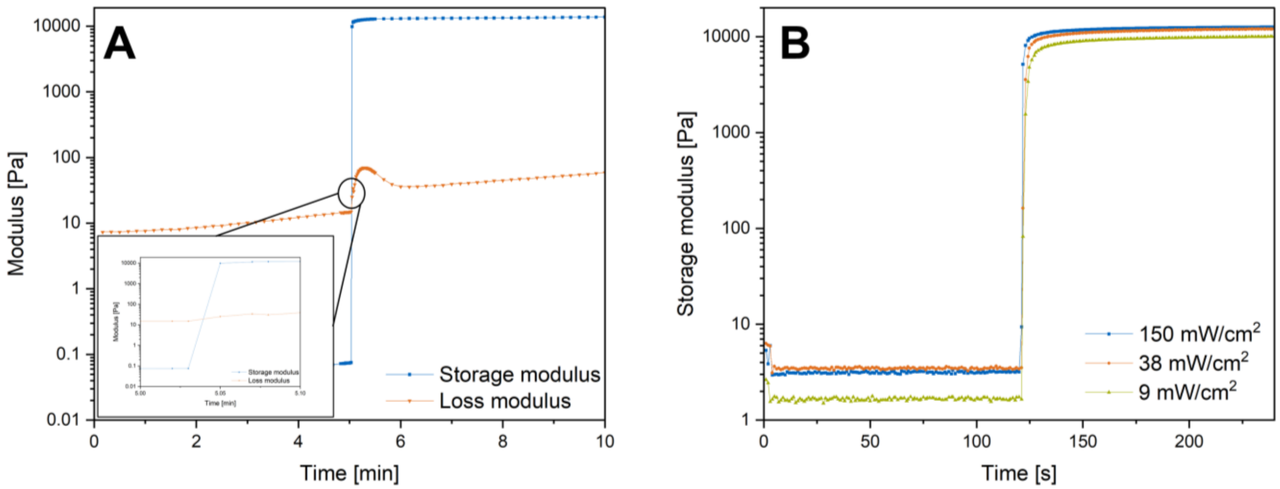

Figure S20: Kinetic investigation of the photochemical crosslinking of P(DEVP-stat-DAlVP) (Table 1, Entry 1) via photoinitiated thiol-ene click chemistry. Behavior of G' and G'' over time (γ = 1%, f = 5 Hz) with irradiation (λ = 365 nm) after 5 minutes and a shorter measurement interval (t = 1 s) in the range of the sol-gel transition (**A**). Effect of the light intensity on the reaction rate of the thiol-ene reaction visualized by the development of the storage moduli upon crosslinking (light source activated after 120 seconds) (**B**).

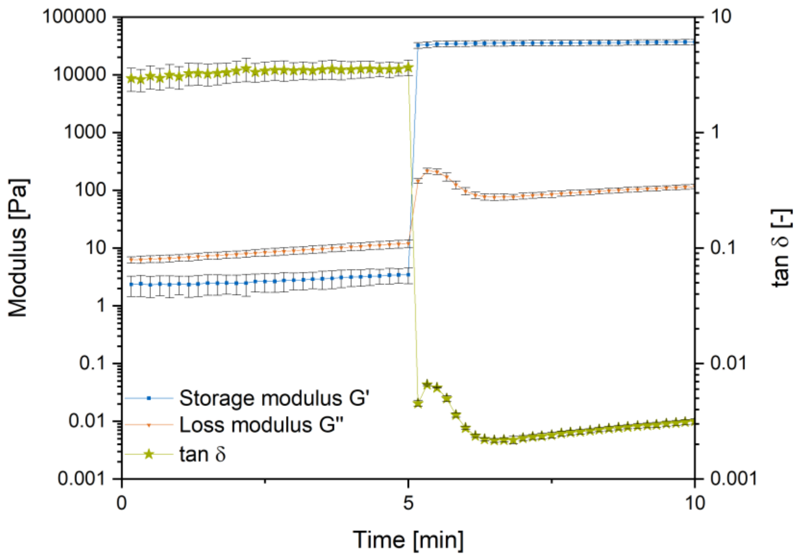

Figure S21: Rheological investigation of gelation process: behavior of G’, G’’ and tan δ over time (γ = 1%, f = 5 Hz) with irradiation (λ = 365 nm) after 5 minutes.

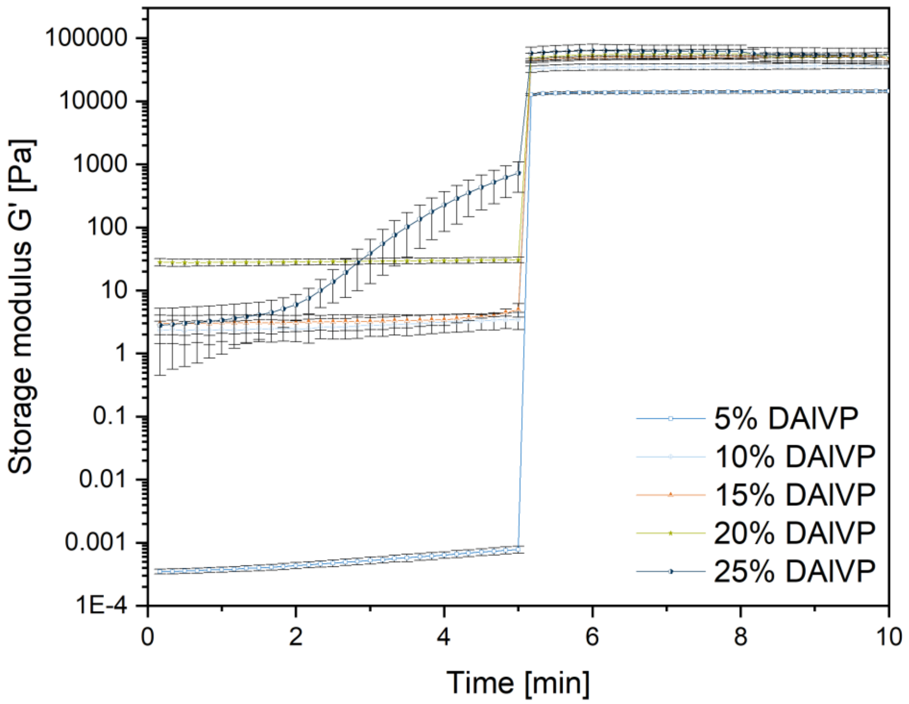

Figure S22: Behavior of the storage modulus over time and standard deviation for the evaluation of the mechanical strength of hydrogels formed from different P(DEVP-stat-DAlVP) copolymers at standard measurement conditions (γ = 1%, f = 5 Hz). Start of irradiation (λ = 365 nm) after 5 minutes to give the plateau values of G' displayed in Figure 2A of the manuscript.

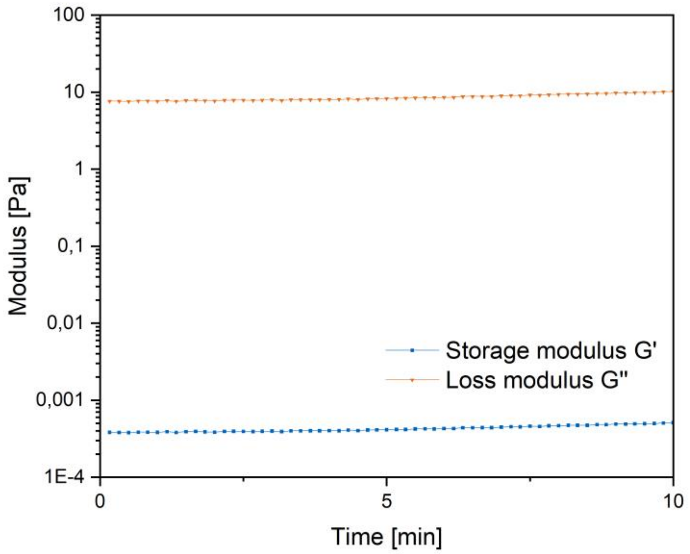

Figure S23: Rheological control experiment at standard measurement conditions (γ = 1%, f = 5 Hz) without irradiation of the sample.

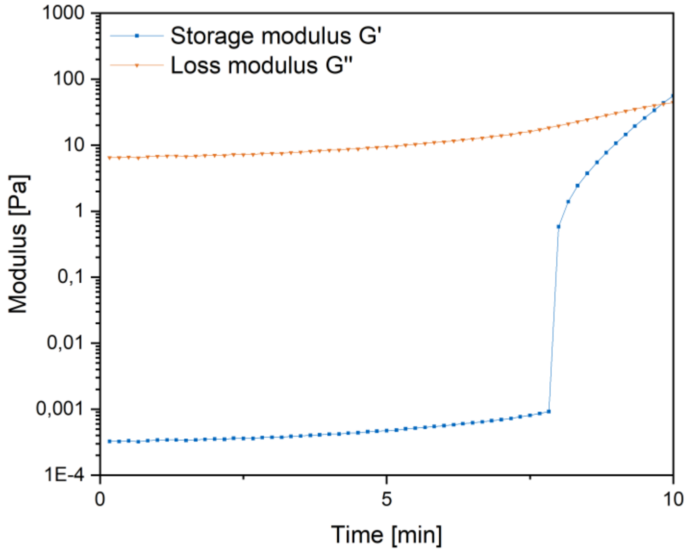

Figure S24: Rheological control experiment at standard measurement conditions (γ = 1%, f = 5 Hz) with irradiation after 5 minutes and no initiator.

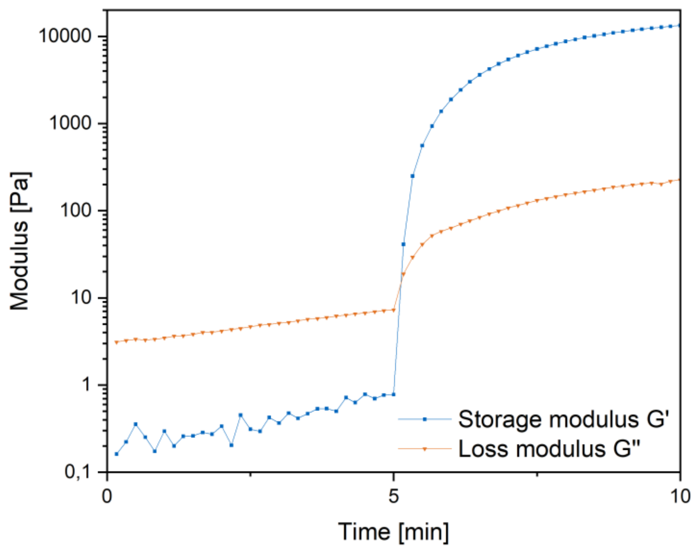

Figure S25: Rheological control experiment at standard measurement conditions ($\gamma = 1\%$, f = 5 Hz) with irradiation after 5 minutes without crosslinker.

Hydrogel synthesis for swelling experiments

To obtain crosslinked polymers for swelling experiments, 100 mg of respective P(DEVP-stat-DAlVP) copolymer were dissolved in 0.3 mL of dioxane in a polypropylene cup using a vortex mixer. Subsequently, calculated amounts of 3,6-dioxa-1,8-octanedithiol (2.50 eq. with respect to the allyl groups of the polymer) and 2,2-dimethoxy-2-phenylacetophenone (0.40 eq. with respect to the allyl groups of the polymer) were added under light exclusion. Then, the mixture was homogenized by vortexing and subjected to UV-irradiation ($\lambda = 365$ nm) for 60 minutes. Further, the solvent-swollen hydrogel chips obtained with this were dried in vacuo ($p = 6 \cdot 10^{-3}$ mbar) for 16 hours to remove residual solvent and excess crosslinker. Finally, after determining the weight of the dry samples, the dry gels were immersed in distilled water and swollen to constant mass for approximately 7 hours. To quantify the water uptake, the hydrogels were removed from the

solvent, and excess water was removed using paper tissue. The swelling ratio was calculated by comparing the weight of the swollen sample and the weight of the dry sample, as described in the manuscript.

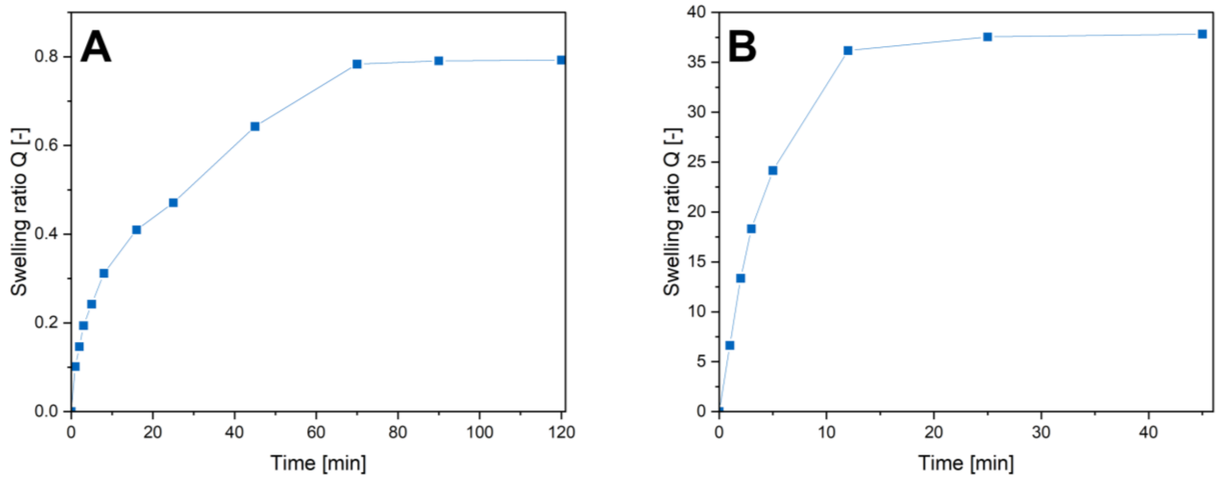

Figure S26: Water uptake kinetics of hydrogels originating from an unfunctionalized polymer (Table 1, Entry 4) (**A**) and a sulfonate-modified polymer (Table 2, Entry 11) (**B**).

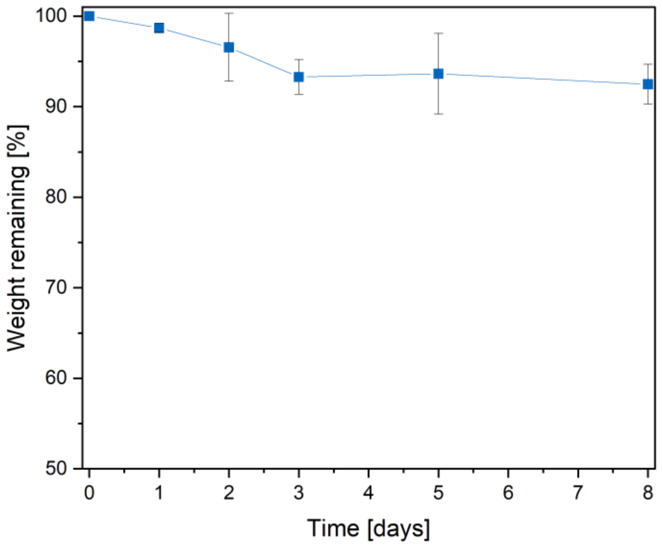

Figure S27: Investigation of the hydrolytic stability of hydrogels synthesized from Entry 1 (Table 1) under physiological conditions by determining the remaining weight relative to the weight of the swollen state over eight days in phosphate-buffered saline solution (pH 7.4) at 37 °C.

Functionalization of P(DEVP-stat-DAlVP) copolymers with sodium 3-mercaptopropane-1-sulfonate

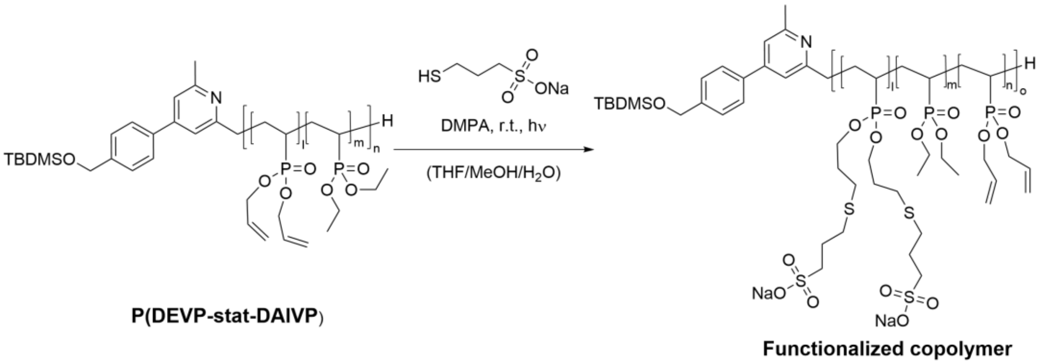

(2)

The functionalization of P(DEVP-stat-DAlVP) with sodium 3-mercaptopropane-1-sulfonate was carried out according to a literature-known procedure.[1] In an oven-dried Schlenk flask, P(DEVP-

stat-DAlVP) was dissolved in a mixture of tetrahydrofuran, methanol, and water (THF/MeOH/H_2O = 5/1/1) (10 mL solvent per 100 mg of polymer). Subsequently, the calculated amounts of sodium 3-mercaptopropane-1-sulfonate (0.30 eq. with respect to the allyl groups of the polymer) and 2,2-dimethoxy-2-phenylacetophenone (DMPA) (0.10 eq. with respect to the allyl groups of the polymer) were added. The resulting clear solution was degassed through repeated application of vacuum and flooding with argon (15 iterations) and irradiated (λ = 365 nm) at room temperature for 15 hours. After the photoreaction, the solvent was removed in vacuo, and the residue was taken up in small amounts of distilled water. The functionalized polymer was then purified via dialysis (MWCO = 8 kDa) against distilled water and finally freeze-dried to afford the pure, functionalized polymers.

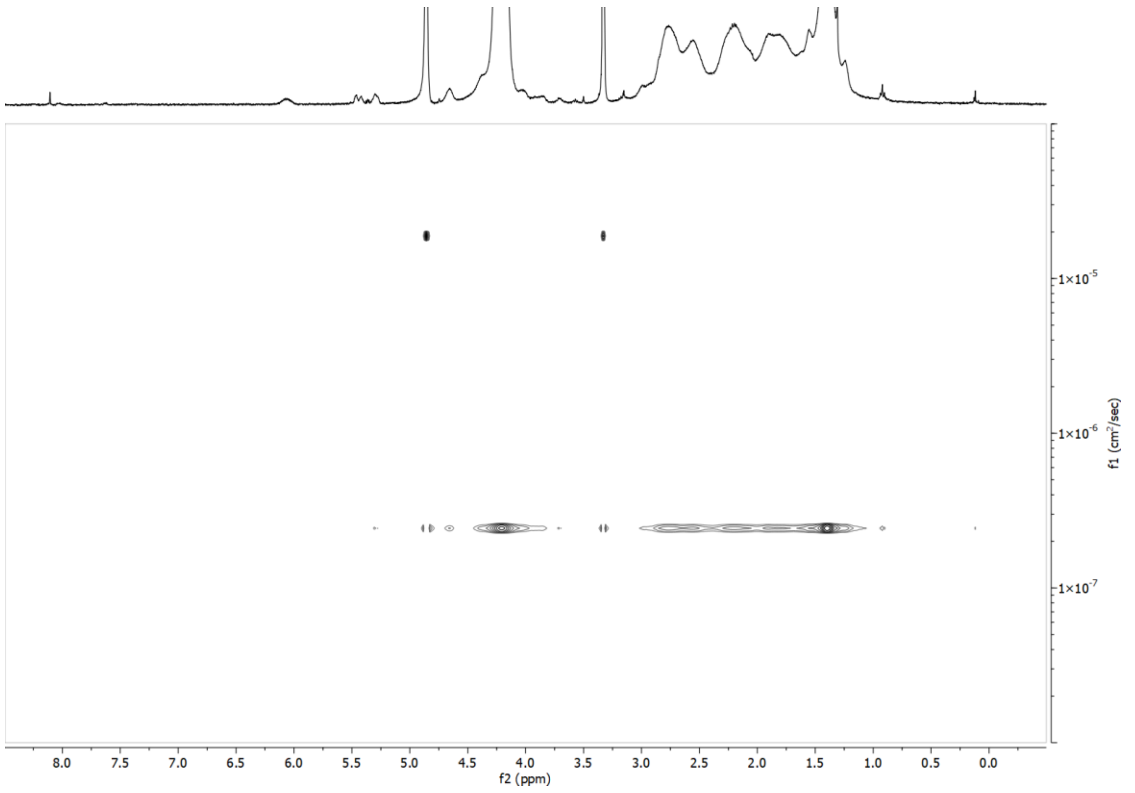

Figure S28: Exemplary ^{1}H DOSY NMR spectrum of functionalized P(DEVP-stat-DAlVP) in MeOD (Table 2, Entry 9).

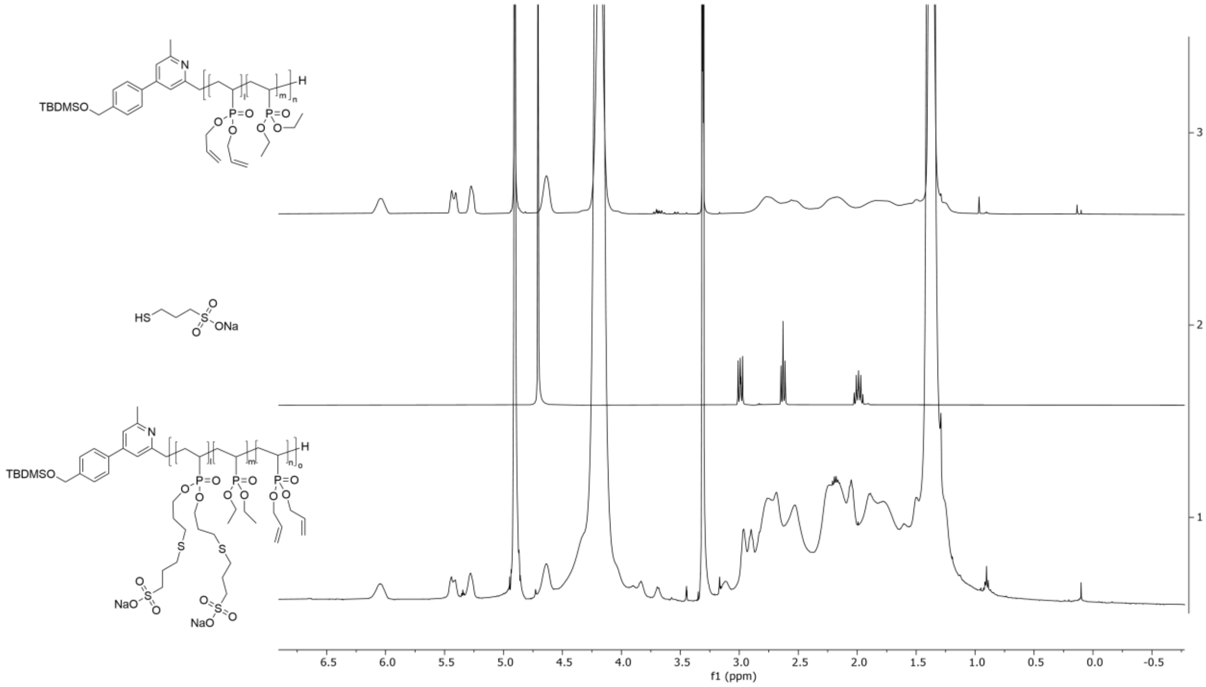

Figure S29: Functionalization of P(DEVP-stat-DAlVP) with sodium 3-mercaptopropane-1-sulfonate: ^{1}H-NMR spectrum of P(DEVP-stat-DAlVP) in MeOD (top), ^{1}H-NMR spectrum of sodium 3-mercaptopropane-1-sulfonate in D_2O (middle) and ^{1}H-NMR spectrum of functionalized P(DEVP-stat-DAlVP) (Table 2, Entry 10) in MeOD (bottom).

Table S2: Copolymer compositions determined via ^{1}H-NMR spectroscopy prior to click reaction and results of elemental analysis of functionalized polymers representing the calculation basis for the polymer compositions in the functionalization of P(DEVP-stat-DAlVP) with sodium 3-mercaptopropane-1-sulfonate.

Entry	DEVP unfunctionalized polymer [%]	DAlVP unfunctionalized polymer [%]	Equivalents of sulfonate with respect to allyl groups [-]	Theoretical sulfur content [wt.%]	Sulfur weight percentage via elemental analysis [wt.%]	Percentage of targeted functionalization degree of allyl groups [%]
9	96.2	3.8	0.30	0.87	0.53	60
10	90.1	9.9	0.30	2.15	1.51	70
11	84.7	15.3	0.30	3.19	2.72	85
12	79.4	20.6	0.30	4.15	2.74	66

Hydrogel synthesis and swelling with functionalized P(DEVP-stat-DAlVP)

The synthesis of hydrogels starting from functionalized P(DEVP-stat-DAlVP) was carried out similarly as described above. Unlike for the unfunctionalized copolymers, additional small amounts of water were added for polymers with high degrees of functionalization to help with solubilization of these highly hydrophilic compounds and achieve homogenous solutions for

crosslinking. The crosslinking and the determination of the water uptake were performed in the same way as for unfunctionalized polymers.

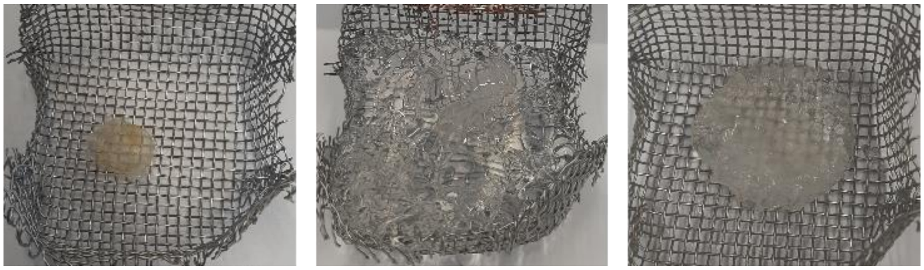

Figure S30: Images of hydrogel samples obtained from functionalized polymers: hydrogel in the dry state (left), highly swollen hydrogel without structural integrity (Table 2, Entry 9) (middle) and swollen hydrogel forming a soft and brittle chip (Table 2, Entry 11).

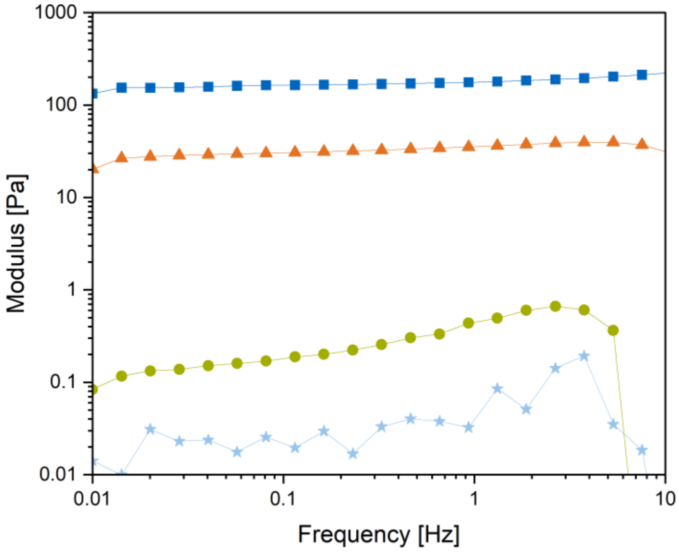

Figure S31: Frequency sweeps on fully gelled samples synthesized from sulfonate-functionalized polymers (83 mg mL^{-1}) with a deformation of γ = 1% and frequencies between 0.01 and 10 Hz: Entry 9 (light blue), entry 10 (green), entry 11 (orange), and entry 12 (blue).

4. HYDROGEL PURIFICATION AND CYTOTOXICITY TESTING

Hydrogel purification by Soxhlet extraction

Table S3: Sample descriptions, purification procedures, and fractions of extractable compounds and gel for hydrogels used in the extract test performed according to ISO 10993.

Hydrogel	Description	Solvent	Temperature (distillation flask)	Duration	Extractable fraction [%]	Gel Fraction [%]
1	P(DEVP-stat-DAlVP) (5% DAlVP), standard crosslinker (excess during synthesis)	EtOH, H_2O	140 °C (EtOH)<br>150 °C (H_2O)	9 h (EtOH)<br>18 h (water)	23	77
2	P(DEVP-stat-DAlVP) (5% DAlVP), standard crosslinker (excess during synthesis)	EtOH, H_2O	140 °C (EtOH)<br>150 °C (H_2O)	9 h (EtOH)<br>18 h (water)	14	86
3	P(DEVP-stat-DAlVP) (5% DAlVP), standard crosslinker (stoichiometric amounts)	EtOH, H_2O	140 °C (EtOH)<br>150 °C (H_2O)	9 h (EtOH)<br>18 h (water)	13	87
4	P(DEVP-stat-DAlVP) (10% DAlVP), standard crosslinker (excess during synthesis)	H_2O	150 °C	25 h	33	67
5	P(DEVP-stat-DAlVP) (10% DAlVP) standard crosslinker (stoichiometric amounts)	H_2O	150 °C	25 h	26	74

In the first step, the weight of dry samples subjected to Soxhlet extraction was determined for the calculation of sol and gel fractions. Subsequently, the dry gels were transferred into the thimble, and the Soxhlet apparatus was assembled. Soxhlet extraction was either carried out with ethanol followed by water or with water exclusively (for experimental details, see Table S3). After

complete purification, the hydrogels were dried in vacuo ($p = 6 \cdot 10^{-3}$ mbar) for 16 hours and the weight determined for comparison with the initial weight.

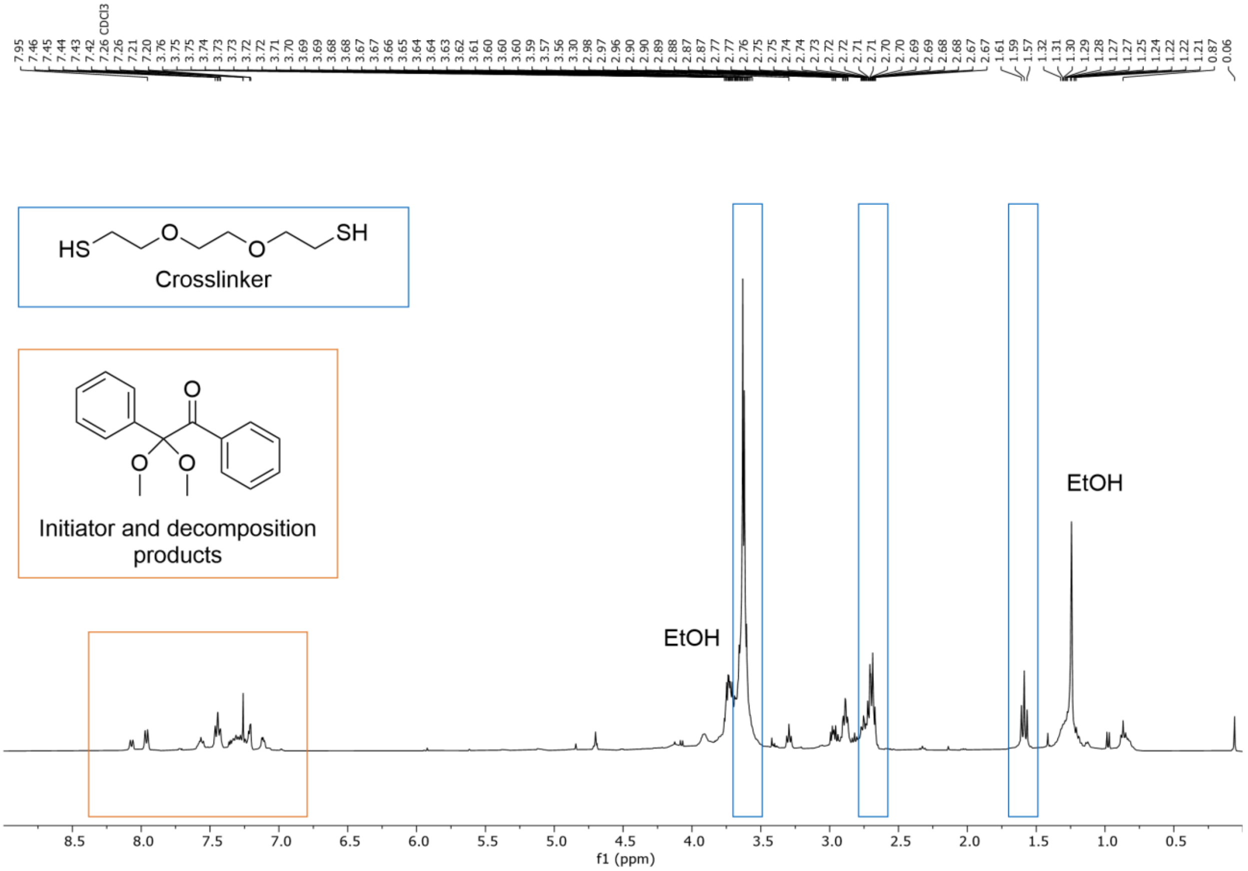

Figure S32: ^{1}H-NMR spectrum (solvent: $CDCl_3$) of the residue in the distillation flask after removal of the solvent from the Soxhlet extraction of Hydrogel 2, Table S2.

Cytotoxicity test

The cytotoxicity testing of hydrogels was conducted based on the extract test according to the ISO 10993. Human umbilical artery smooth muscle cells (HUASMCs) were cultured in Dulbecco's modified Eagle medium (DMEM, Gibco) supplemented with 10% v/v fetal bovine serum (FBS, Gibco) and 1% antibiotics/antimycotics (ABM, Gibco) at 37°C in a humidified atmosphere containing 5% CO_2. The developed hydrogels were first washed twice using 70% ethanol for 30 minutes each, subsequently rinsed with phosphate-buffered saline solution (PBS, Gibco), and then incubated in DMEM for 72 h at 37°C to create the eluates according to the defined weight/volume extraction ratios defined by the ISO. Untreated DMEM (Gibco, USA) was used as the negative control, and the eluate of a latex glove was incubated in DMEM for 72 h at 37°C as the positive control. HUASMCs were cultured in untreated medium in 96 wells at a cell density of 10 000 cells cm^{-2} for 24 h, then the medium was aspirated before the eluates of the samples and controls were transferred to the corresponding wells. After 72 hours at 37°C and 5 % CO_2, the XTT proliferation assay (Invitrogen) was performed according to the manufacturer's instructions, and the absorbance at 450 nm was measured using the microplate reader (Spark, Tecan). The relative absorbance was calculated by normalizing the absorbance values to that of the negative control. Hydrogels exhibiting cell viability above 70 %, which is the threshold defined by the ISO, were considered non-cytotoxic.

Cell adhesion test

The hydrogels were washed with 70 % ethanol for 1 hour, rinsed with phosphate-buffered saline solution (PBS, Gibco), dried for 20 minutes, and transferred into 96 well plates (Greiner Bio-One). Human umbilical artery smooth muscle cells (HUASMCs) were seeded at a density of

10 000 cells cm^{-2} on the hydrogel surface and incubated in DMEM (Gibco, USA), supplemented with 10 % fetal bovine serum (Gibco, USA) and 1 % antibiotic-antimycotic (Gibco, USA) for 72 hours. The surface of a well plate (Greiner Bio, Germany) was used as the control. The XTT proliferation assay (Invitrogen) was performed according to the manufacturer's instructions, and the absorbance at 450 nm was measured using the microplate reader (Spark, Tecan). The relative absorbance was calculated by normalizing the absorbance values to that of the control (well surface). Subsequently, the cells were fixed in a 4 % formaldehyde solution (Carl Roth, Germany) in PBS for 15 minutes at room temperature and stained for fluorescence imaging. Briefly, the samples were blocked and permeabilized with PBS containing 5 % normal goat serum (Dako, Germany) and 0.1 % Triton X-100 (Sigma, Germany) for 60 min at room temperature. Staining for actin was performed with Acti-stain phalloidin 488 (Biozol, Germany) for 1 hour at 37 °C, and nuclei staining subsequently with DAPI (Invitrogen, USA) for 5 minutes at room temperature. Fluorescence images were taken with the BZ-X800 microscope (Keyence, Japan).

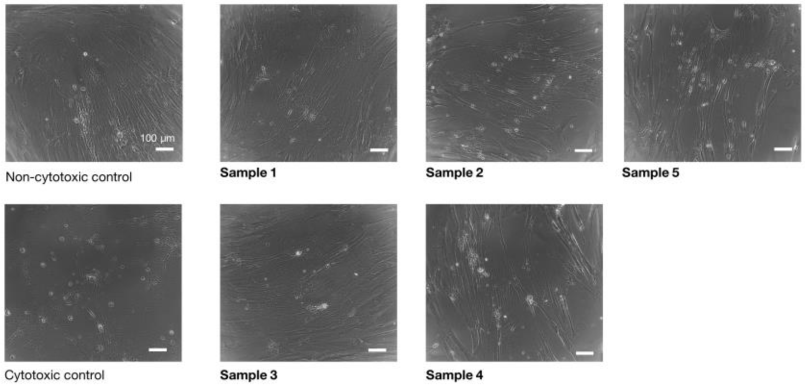

Figure S33: Qualitative evaluation of the cytotoxicity test based on the extract test according to ISO 10993. Microscopic images of human umbilical artery smooth muscle cells (HUASMCs) incubated for 72 h in presence of the eluates of Hydrogels 1-5 as well as of untreated cell culture medium as the negative control (NC) and of the eluate of a latex glove as the positive control (PC). Scale bar: 100 µm.

5. ADDITIONAL BIOCOMPATIBILITY STUDIES

Immune response via ELISA THP-1 cytokine release

The immune response to the hydrogels was evaluated by quantifying the cytokine release of monocyte-derived macrophages. The human cell line THP-1 (ATCC) was cultured in RPMI 1640 medium (Gibco, Life Technologies, Paisely, UK) supplemented with 10% fetal calf serum (Gibco), 2 mM l-glutamine (Gibco), 1 mM sodium pyruvate (Gibco), 1 U mL^{-1} penicillin and 1 µg mL^{-1} streptomycin (Gibco). The cells were differentiated into M0 macrophages using 100 µm phorbol 12-myristate 13-acetate for 72 h and were allowed to rest in a complete culture medium for 24 h. Prior to the assay, circular hydrogel discs with a surface area of 1 cm^2 were incubated in RPMI medium overnight to reach an equilibrium in their liquid uptake. The cells were then seeded at a density of 250 000 cells cm^{-1} onto the samples, and the released cytokine concentration in the cell culture supernatant was evaluated after 72 h of incubation at 37 °C with 5% CO_2. The released pro-inflammatory (interleukin-6, tumor necrosis factor-α) and anti-inflammatory (transforming growth factor β, interleukin-10) cytokines were quantified with the DuoSet ELISA Development System (R&D Systems, Minneapolis, MN, USA) following the manufacturer's instructions using a multimode microplate reader (Spark, Tecan, Männedorf, Switzerland).

Bacterial adhesion

Determination of the bacterial adhesion was conducted with the strains *S. aureus* NCTC 8325-4 and *E. coli* ATCC 25922. Circular hydrogel discs with a diameter of 6 mm were sterilized in 70% ethanol for 1 h and then washed in phosphate-buffered saline (PBS) for 1 h. 10 µl of the bacterial suspensions with a concentration of 10^9 CFU mL^{-1} were subsequently seeded onto the hydrogel samples. After incubating the samples at 37 °C for 3 h, non-adherent bacteria were removed by

washing the samples thrice in 5 mL sterile PBS. The samples were then vortexed in PBS for 1 min to remove the adherent bacteria, which were quantified by plating serial dilutions on Chapman agar. The agar plates were incubated at 37 °C for 48 h, and the colonies were counted to quantify the number of adherent bacteria. Log reduction is calculated by determining the log10 of the ratio of the initial seeding concentration to the final adherent bacterial concentration.

Endothelialization

To evaluate the ability of the hydrogels to support endothelialization, human umbilical vein endothelial cells (HUVECs) were used to investigate the formation of an endothelial layer on the hydrogel sample surface. HUVECs were cultured in endothelial cell basal medium (EBM-2, Lonza Group AG, Basel, Switzerland) supplemented with the endothelial growth medium 2 kit. Supplements included 0.1% insulin, 0.1% gentamicin, 0.1% ascorbic acid, 0.4% human fibroblast growth factor, 2% fetal bovine serum (FBS), 0.1% endothelial growth factor, 0.04% hydrocortisone, 0.1% epidermal growth factor, and 0.1% heparin. Prior to the assay, circular hydrogel discs with a diameter of 6 mm were incubated in EGM-2 medium overnight to reach an equilibrium in their liquid uptake. The cells were then seeded at a density of 10 000 cells cm^{-1} onto the samples. After incubation for 24 hours at 37 °C, the samples were rinsed with PBS and fixed in 4% paraformaldehyde (Thermo Fisher Scientific Inc., USA) at room temperature for 15 min. Then, the samples were washed three times in PBS and blocked in a PBS solution (Gibco) with 5% normal goat serum (DAKO GmbH, Jena, Germany) for 1 h at room temperature. CD31 primary antibody (Sigma) 1:100 diluted in 0.1 % bovine serum albumin solution (BSA, Sigma, Germany) was added for 1 h at room temperature and followed by three washing steps for 5 min in PBS. Alexa 647 secondary antibody (Molecular Probes) 1:400 diluted in 0.1 % BSA solution was subsequently added. After 1 h incubation at room temperature, the samples were washed 3x

for 5 min in PBS. DAPI diluted in PBS (0.2 µg/ml) was added for nuclei staining (Thermo Fisher Scientific Inc, USA). Finally, the samples were imaged under the fluorescence microscope BZ-X800 (Keyence, Japan).

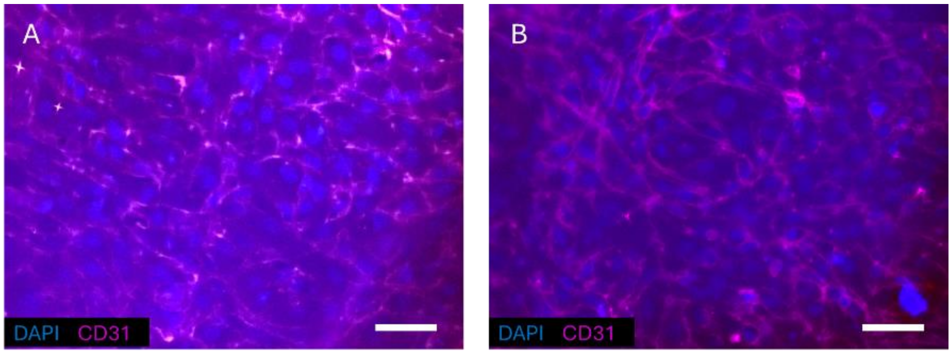

Figure S34: Immunohistochemical analysis of the endothelialization of the hydrogel samples. Immunohistochemical staining for CD31 of seeded endothelial cells on HG1 (**A**) and HG2 (**B**). 4′,6-diamidino-2-phenylindole (DAPI) was used as cell nuclear counterstain (blue). Scale bar: 50 µm.

6. REFERENCES

(1) Halama, K.; Schaffer, A.; Rieger, B. Allyl Group-Containing Polyvinylphosphonates as a Flexible Platform for the Selective Introduction of Functional Groups via Polymer-Analogous Transformations. *RSC Adv.* **2021**, *11* (61), 38555–38564.

(2) Salzinger, S.; Soller, B. S.; Plikhta, A.; Seemann, U. B.; Herdtweck, E.; Rieger, B. Mechanistic Studies on Initiation and Propagation of Rare Earth Metal-Mediated Group Transfer Polymerization of Vinylphosphonates. *J. Am. Chem. Soc.* **2013**, *135* (35), 13030–13040.

(3) Schaffer, A.; Kränzlein, M.; Rieger, B. Synthesis and Application of Functional Group-Bearing Pyridyl-Based Initiators in Rare Earth Metal-Mediated Group Transfer Polymerization. *Macromolecules* **2020**, *53* (11), 4345–4354.

(4) Soller, B. S.; Salzinger, S.; Jandl, C.; Pöthig, A.; Rieger, B. C–H Bond Activation by σ-Bond Metathesis as a Versatile Route toward Highly Efficient Initiators for the Catalytic Precision Polymerization of Polar Monomers. *Organometallics* **2015**, *34* (11), 2703–2706.
